# Multicentre individual randomised controlled trial of screening and brief alcohol intervention to prevent risky drinking in young people aged 14–15 in a high school setting (SIPS JR-HIGH): study protocol

**DOI:** 10.1136/bmjopen-2016-012474

**Published:** 2016-12-23

**Authors:** Emma L Giles, Simon Coulton, Paolo Deluca, Colin Drummond, Denise Howel, Eileen Kaner, Elaine McColl, Ruth McGovern, Stephanie Scott, Elaine Stamp, Harry Sumnall, Les Tate, Liz Todd, Luke Vale, Viviana Albani, Sadie Boniface, Jennifer Ferguson, Jo Frankham, Eilish Gilvarry, Nadine Hendrie, Nicola Howe, Grant J McGeechan, Grant Stanley, Dorothy Newbury-Birch

**Affiliations:** 1 Health and Social Care Institute, Alcohol and Public Health Team, Teesside University, Middlesbrough, UK; 2 Centre for Health Services Research, George Allen Wing, University of Kent, Canterbury, UK; 3 Addictions Department, Institute of Psychiatry, Psychology & Neuroscience, King's College London, London, UK; 4 Institute of Health and Society, Newcastle University, Newcastle upon Tyne, UK; 5 Newcastle Clinical Trials Unit, Newcastle University, Newcastle upon Tyne, UK; 6 Centre for Public Health, Liverpool John Moores University, Liverpool, UK; 7 Young People's Drug and Alcohol Department, North Tyneside Council, Tyne and Wear, UK; 8 School of Education, Communication and Language Sciences, Newcastle University, Newcastle upon Tyne, UK; 9 Health Economics Group, Institute of Health and Society, Newcastle University, Newcastle upon Tyne, UK; 10 Faculty of Education, Health and Community, Liverpool John Moores University, Liverpool, UK; 11 Northumberland, Tyne and Wear NHS Foundation Trust, St. Nicholas Hospital, Newcastle upon Tyne, UK

**Keywords:** Alcohol, Brief Intervention, Randomised Controlled Trial, School Setting

## Abstract

**Introduction:**

Drinking has adverse impacts on health, well-being, education and social outcomes for adolescents. Adolescents in England are among the heaviest drinkers in Europe. Recently, the proportion of adolescents who drink alcohol has fallen, although consumption among those who do drink has actually increased. This trial seeks to investigate how effective and efficient an alcohol brief intervention is with 11–15 years olds to encourage lower alcohol consumption.

**Methods and analysis:**

This is an individually randomised two-armed trial incorporating a control arm of usual school-based practice and a leaflet on a healthy lifestyle (excl. alcohol), and an intervention arm that combines usual practice with a 30 min brief intervention delivered by school learning mentors and a leaflet on alcohol. At least 30 schools will be recruited from four regions in England (North East, North West, London, Kent and Medway) to follow-up 235 per arm. The primary outcome is total alcohol consumed in the last 28 days, using the 28 day Timeline Follow Back questionnaire measured at the 12-month follow-up. The analysis of the intervention will consider effectiveness and cost-effectiveness. A qualitative study will explore, via 1:1 in-depth interviews with (n=80) parents, young people and school staff, intervention experience, intervention fidelity and acceptability issues, using thematic narrative synthesis to report qualitative data.

**Ethics and dissemination:**

Ethical approval was granted by Teesside University. Dissemination plans include academic publications, conference presentations, disseminating to local and national education departments and the wider public health community, including via Fuse, and engaging with school staff and young people to comment on whether and how the project can be improved.

**Trial registration trial:**

ISRCTN45691494; Pre-results.

**Strengths and limitations of this study**

- A robust randomised controlled study design.
- Validated screening tools used to measure attitudes and behaviours.
- Limited prior research has explored the use of alcohol brief interventions in UK school settings.
- This definitive trial follows on from a successful pilot feasibility trial.
- The study relies on recruitment of sufficient school sites and willingness of learning mentors to engage with the trial.

## Background

Adolescents in England are among the heaviest drinkers in Europe.^1^ The percentage of young people who have ever had an alcoholic drink in England increases with age from 10% of adolescents aged 11–12 years to 34% of adolescents aged 13–15 years, and the prevalence of drinking in the last week rises from 1% of 11year olds to 18% of 15year olds making them an important age group to target.^2^ In recent years, the proportion of adolescents who drink alcohol has fallen, although consumption among those who do drink has actually increased.^2^ Alcohol can have adverse impacts on health, well-being, education and social (including learning) outcomes for many young people who are drinking alcohol. The impact of alcohol on the development and behaviour of young people has been well researched in early,^3^ middle^4^ and late adolescence.^5^ It is now well known that young people are much more vulnerable than adults to the adverse effects of alcohol, due to a range of physical and psycho-social factors which often interact.^6^

There is no standardised definition of risky drinking in young people and so our definition encompasses commonly understood concepts of hazardous drinking (at a level or pattern that increases the risk of physical or psychological problems), harmful drinking (defined by the presence of these problems) and binge drinking (risky single occasion, high-intensity drinking which can be episodic) as well as the Department of Health concepts of increasing and high-risk drinking.^7^ The Chief Medical Officer for England has provided recommendations on alcohol consumption in young people^8^ based on an evidence review of the risks and harms of alcohol to young people.^6^ The recommendations state that children should abstain from alcohol before the age of 15 and those aged 15–17 are advised not to drink, but if they do drink it should be no more than what equates to adult daily benchmarks.^9^

### Primary and secondary preventative interventions for risky drinking

There is a large volume of research on universal prevention to reduce risky drinking in the school setting.^10^ ^11^ Such prevention is directed at all young people, whether they drink alcohol or not, and aims to delay the age that drinking begins, often via general health education. This body of work has shown mixed results with only a small number of programmes reporting that interventions delivered in a school setting were more effective in reducing alcohol use than control conditions.^12^ Secondary prevention, that is, targeting interventions at young people who are already drinking alcohol, may be a more effective and efficient strategy since the intervention is likely to have more salience for the individuals receiving it.^13^ ^14^

This secondary prevention generally consists of alcohol brief interventions and screening (to identify relevant potential recipients) followed by structured advice or counselling of short duration which is aimed at reducing alcohol consumption or decreasing problems associated with drinking.^15^ The interventions are often based on social cognitive theory which is derived from social learning theory.^16^ These types of intervention have been found to be particularly effective with this age group.^13^

This current research aims to develop the evidence base by focusing on a secondary prevention intervention of screening and brief intervention to reduce risky drinking in younger adolescents (aged 14–15) in a school context. The study follows on from the SIPS JR-HIGH pilot feasibility study which was funded by the National Institute of Health Research Public Health Programme (NIHR PHR) (ISRCTN07073105).^14^

## Main trial

### Aims, objectives and methods

#### Research aim

The aim of the study is to evaluate the effectiveness and cost-effectiveness^i^ of alcohol screening and brief intervention to reduce risky drinking in young people aged 14–15 in the English school setting. Validated tools will be used in the study for primary and secondary outcomes measures.

#### Primary outcome

- The primary outcome of the trial is total alcohol consumed in standard units in the last 28 days, using the 28 day Timeline Follow Back questionnaire^17^ at 12-month follow-up.

#### Baseline secondary outcome measurements

- Student Alcohol Questionnaire (A-SAQ)^18^ to measure risky drinking (scoring ‘4 or more times but not every month’, ‘at least once a month but not every week’, ‘every week but not every day’, or ‘every day’);
- Alcohol use frequency, quantity (on a typical occasion) and binge drinking (six or more drinks in one session for men and women)^19^ assessed using the modified 10 question Alcohol Use Disorders Identification Test (AUDIT);^20^ ^21^
- Alcohol-related problems assessed using the validated Rutgers Alcohol Problems Inventory (RAPI) which includes measures on aggression;^22^
- Drunkenness during the last 30 days, dichotomised as ‘never’ and ‘once or more’;^4^
- Drinking motives assessed using the 20-item Drinking Motives Questionnaire (DMQ). This tool uses a six-point Likert scale, which measures motives to drinking across four domains (social, coping, enhancement and conformity). Higher scores within each domain indicate stronger endorsement of positive reinforcement received through consumption of alcohol;^5^
- General psychological health using the 14-item Warwick Edinburgh Mental Well-Being Scale (WEMWBS).^23^ This tool uses a 5-point Likert scale which gives a score of one to five per question giving a minimum score of 14 and maximum score of 70. A higher WEMWBS score indicates a higher level of mental well-being;^24^ ^25^
- Two questions relating to sexual risk taking are included. These are the same questions as in the pilot study.^14^ These questions are: ‘After drinking alcohol, have you engaged in sexual intercourse that you regretted the next day?’ and ‘After drinking alcohol, have you ever engaged in sexual intercourse without a condom?’ Both questions can be answered with one of the three following options: ‘I have never engaged in sexual intercourse’, ‘Yes’, or ‘No’;
- Energy drink consumption will be assessed by asking young people how many times a week they consume energy drinks. Young people can answer: ‘never’, ‘less than once a week’, ‘2–4 days a week’, ‘5–6 days a week’, ‘every day once a day’ and ‘every day more than once a day’;
- Age of first smoking and how many cigarettes were smoked in the past 30 days;^1^
- Demographic information collected will include gender and ethnicity. The first part of the postcode will be collected for trial participants;
- Quality of life measured using the EQ-5D Y, which is a valid measure for those aged 12 or older, and will be used to measure health-related quality of life.^26^ Responses to the five items will be converted into utility scores using the UK population algorithm. This will be administered at baseline and 12 months post intervention;^26^
- Quality-adjusted life years (QALY) estimated using general population tariffs from responses to EQ-5D Y administered and scored at baseline and 12 months.

#### 12-Month follow-up measurements

- All tools assessed at baseline;
- Per cent days abstinence over last 28 days, drinks per drinking day and days>2 units from 28 day TLFB;
- Incremental cost per QALY gained at 12 months;
- Depending on findings, modelled estimates of incremental cost per QALY and cost-consequences in the longer term;
- National Health Service (NHS), educational, social and criminal services data estimated using a modified S-SUQ^27^ and a learning mentor case diary developed in the pilot study, measured at 12 months post intervention;
- Cost-consequences presented in the form of a balance sheet for outcomes at 12 months. (For further details, see online supplementary appendix 1).

10.1136/bmjopen-2016-012474.supp1 supplementary appendix

### Trial participants

Young people aged 14–15 years in Year 10 in at least 30 Secondary/High schools/Academies in four areas: the North East of England, North West of England, Kent and Medway, and London. Schools will be included if they have learning mentors (or equivalent members of pastoral staff, including teachers fulfilling this role) employed by the school (most schools have these pastoral/learning mentor staff roles). Screening will take place in the personal, social and health education (PSHE) or equivalent lesson, registration class, on a classroom basis, or in assembly. Interventions will take place in the learning mentor's classroom or office space. Pupils receive minimal recompense for taking part in the trial (an ‘admit one’ cinema voucher), and each participating school will receive £1000 to assist with administration and other costs of full research participation.

#### Inclusion criteria

Young people aged 14–15 years inclusive, whose parents do not opt them out of the study, scoring positively on the A-SAQ, leaving their name, and are willing and able to provide informed written assent (see online supplementary appendix 2) for intervention and follow-up.

10.1136/bmjopen-2016-012474.supp2 supplementary appendix

#### Exclusion criteria

Young people already seeking or receiving help for an alcohol use disorder, with a recognised diagnosis of a mental health disorder, or exhibit challenging behaviour.

### Trial procedures

Learning mentors (or equivalent members of pastoral/trained staff; hereafter referred to as ‘learning mentors’) employed by schools will deliver the intervention. All learning mentors will receive school-based training in the study procedures and intervention. Training for learning mentors will be carried out by the trained Research Coordinators. Simulated scenarios between learning mentors will be audio-recorded and learning mentors will be assessed by an interventionist prior to embarking on the study with more training support offered if needed. Learning mentors will be provided with materials and on-going guidance and supervision will be provided by research staff. Support on implementing screening and paperwork relevant to the research will be provided by the research team, with a Research Coordinator in each geographical site. Research staff and trainers will maintain regular contact with schools throughout the study period, including site visits and telephone and email support.

#### Control arm

Usual practice on alcohol health education as delivered normally to all students, including in PSHE lessons and curriculum delivered by class teachers, and usual individualised support for young people with an identified alcohol concern. Young people in the control arm will also be given a healthy lifestyle information leaflet (not containing advice about alcohol) with local sources of help, by the trained staff. Usual practice may vary from school to school and information related to this will be captured by researchers at both time points of the study.

#### Intervention

In addition to input equivalent to the control arm, young people who are eligible and assent to participate will take part in a single 30 min personalised interactive worksheet-based session that was developed during the pilot feasibility trial. This brief intervention is grounded in psychological theory and broadly based on social learning theory which views behaviour as a dynamic interaction between the individual, behaviour and environment. As such, the intervention focuses on personal and contextual factors related to drinking behaviour.^16^ This will be delivered by the trained staff (at school) and will contain personalised feedback about the individual student's drinking behaviour, and behaviour change counselling which encompasses the elements of the FRAMES approach and helps the young person to talk through: how much they drink; how many units are in their drinks; who with, where and why they drink; when they might feel at risk from drinking; what they think are the positive and negatives to drinking; what they perceive others to think about their drinking; whether they would reduce their drinking, why and why not; and what they could do about their drinking (20). The intervention also includes advice about the health and social consequences of continued risky alcohol consumption and a leaflet on alcohol.

#### Randomisation

Neither the learning mentor nor the young person will know which arm they are randomised to until after they assent to take part in the trial. Young people will be individually randomised in a 1:1 ratio to the intervention and control arms. A statistician not otherwise involved with the study will produce a computer-generated allocation list to ensure allocation concealment. All efforts will be made to conceal allocation to young people, school staff (except LM delivering the sessions) and research staff, but we are unable to guarantee that young adults will not discuss their allocation with each other.

### Safeguarding

Should issues arise that concern learning mentors or research staff (eg, alcohol and mental health issues), confidentiality will be broken and the necessary support provided to the young person. Safeguarding issues will be recorded in the trial database. Confidentiality will not be broken otherwise, and school staff will be informed through training that they must work to the same rules as doctors and nurses, meaning that confidentiality can only be broken without consent in exceptional (safeguarding) circumstances. Research/trial staff will provide assistance and support on this issue throughout the trial.

### Recruitment, assent and screening

#### Recruitment

In each of the four geographical sites, initially school performance league tables^28^ will be reviewed and schools from the top, middle and bottom of the league table will be contacted. Snowball sampling will allow contact with potential schools via relationships with recruited schools, although may bias the sample and reduce generalisability of results. That said, efforts will be made to recruit a cross-section of schools, including academy schools, schools in deprived areas and religious schools. Gatekeepers and key personnel (eg, School Board of Governors; County Council contacts) will be approached to suggest—and create an initial contact—with potential schools.

#### Option to opt-out of screening (assent)

In advance of screening, all parents/caregivers (hereafter referred to as ‘parents’) will be informed by letter, sent by the school, that young people will be screened as part of the study within their child's school. Parents will have the choice to opt their child out of the study by completing an opt-out form and sending this (in the freepost envelope provided) to the coordinating research centre at Teesside University. Those young people whose parents have opted them out of the study will not complete the questionnaire. If the opt-out is received after the questionnaire is completed, the questionnaire will be removed from the trial. Additionally, where possible, young people who have been opted-out will not be in the classroom at the time the questionnaire takes place. Obtaining assent to take part in this manner is a method widely used in various national youth questionnaires of alcohol consumption and other health behaviours.^29^

#### Screening for the trial

A video-clip will be played to the young people opted into the study, in each school, to give instructions on completing the questionnaires (see: https://www.youtube.com/watch?v=2ZBm3VZVtx0&feature=em-upload_owner). This video-clip will only provide guidance on the process of questionnaire completion and not on the content. Young people will be asked to voluntarily leave their name and class on the questionnaire. Young people will have the option to: (a) not complete the questionnaire (indicative of lack of assent to screening from the young person); (b) complete the questionnaire anonymously; and (c) complete the questionnaire adding their name and class. Each young person will place their completed questionnaire in an envelope and then return it to the teacher. Teachers will not open these envelopes. Individual responses will not be shared with the class teacher or learning mentor. The Research Coordinator will collect the sealed envelopes from the school. Those young people who have screened positively on the A-SAQ (see below) and have left their name will be eligible for the trial. Completed baseline questionnaires by trial participants will be used for the baseline measurements.

### Data collection

#### Baseline data collection

The study envelope will contain a series of questionnaires, including the study screening questionnaire (part of the A-SAQ): ‘In the last 12 months how often have you drunk more than 3 units of alcohol?’ with the response options of ‘Never’; ‘less than 4 times’; ‘4 or more times but not every month’; ‘at least once a month but not every week’; ‘every week but not every day’; ‘every day’. Scoring ‘4 or more times’, or more frequently, indicates a positive screen and eligibility for the trial. This score was shown in our pilot feasibility trial to be a methodologically robust approach to identifying the adolescent population who may benefit from an intervention.^14^ The A-SAQ will be embedded within a larger questionnaire with items addressing a number of health and lifestyle topics (described above).

#### Invitation to meet with learning mentors

Completed baseline questionnaires will be enclosed in a sealed envelope and returned to the individual universities coordinating each study site. The A-SAQ will be scored and a list of ID numbers and names of those who score positive will be sent to a researcher at Teesside University. After learning mentor training (convened by study site coordinators), learning mentors will be given case packs for each eligible young person. Learning mentors will invite young people who scored positive on A-SAQ on the baseline questionnaire to a meeting with them in their office, where they will open the case pack. In the case pack there will be an information leaflet, a case diary, assent forms and a sealed envelope that contains the randomised condition (intervention or control). Young people will be informed that participation is voluntary and will be given the information leaflet to read before signing the assent form. The assent form also asks for the first part of the young person's postcode. The postcode information will be used to enable a stratified sample of young people to be invited to take part in the qualitative study. Once a young person has assented, the second envelope will be opened which will state whether the participant has been randomised to intervention or control. The learning mentor will then deliver the brief intervention or give the participant the control leaflet (in the same meeting). The completed case packs will be sealed and returned to coordinating sites, then couriered to Teesside University. At all times, only the randomisation statistician (AB), two researchers (JB, LA), and individual learning mentors who meet with the young people will know of the randomisation allocations before the trial ends.

#### 12-Month follow-up

Follow-up will occur 12 months post intervention. All young people who are randomised into the trial will be invited to meet with the Research Coordinator (in school) where they will be asked to complete the same questionnaires used at baseline. The researcher will be blinded to the condition the young person was allocated to. The TLFB (including the primary outcome measure of total alcohol consumption) will also be completed face-to-face in schools with the researcher in order to limit bias in the results. All trial participants will be given an admit-one cinema voucher, to compensate them for their time involved in the study.^30^ Trial participants' baseline and follow-up questionnaires will be linked with a unique ID (the screening number). All participants will be asked by the researcher at follow-up whether they are willing to be contacted for an in-depth interview by a researcher who may be the same individual or another researcher.

#### Intervention fidelity

It is important to ensure learning mentors deliver the intervention in accordance with the intervention manual. To establish intervention fidelity, we will complete competency and fidelity checks at two time points: (1) competency checks of learning mentor training, and (2) fidelity checks of cases delivered during the intervention phase. For the competency checks, each learning mentor will have one simulated intervention with another learning mentor or the research coordinator. Of these sessions, at least 80% will be recorded and ‘signed off’ by an independent expert rater from the research team using the BECCI rating scale.^31^ Additionally, we will attempt to assess 20% of live cases for fidelity (using a pragmatic sampling approach). The BECCI scale is a tool that measures the skills involved in behaviour change counselling. It is scored 0–4 with a score of 2 or more (skills used to ‘some extent’) being acceptable as used in previous studies.^14^ ^32–34^ The young people will provide assent for the live case recording to take place. As the recording and analysis of the delivery of the intervention sessions forms part of the employment contract of the learning mentors, formal consent is not required.

#### Sample size calculation

**Figure 1:**
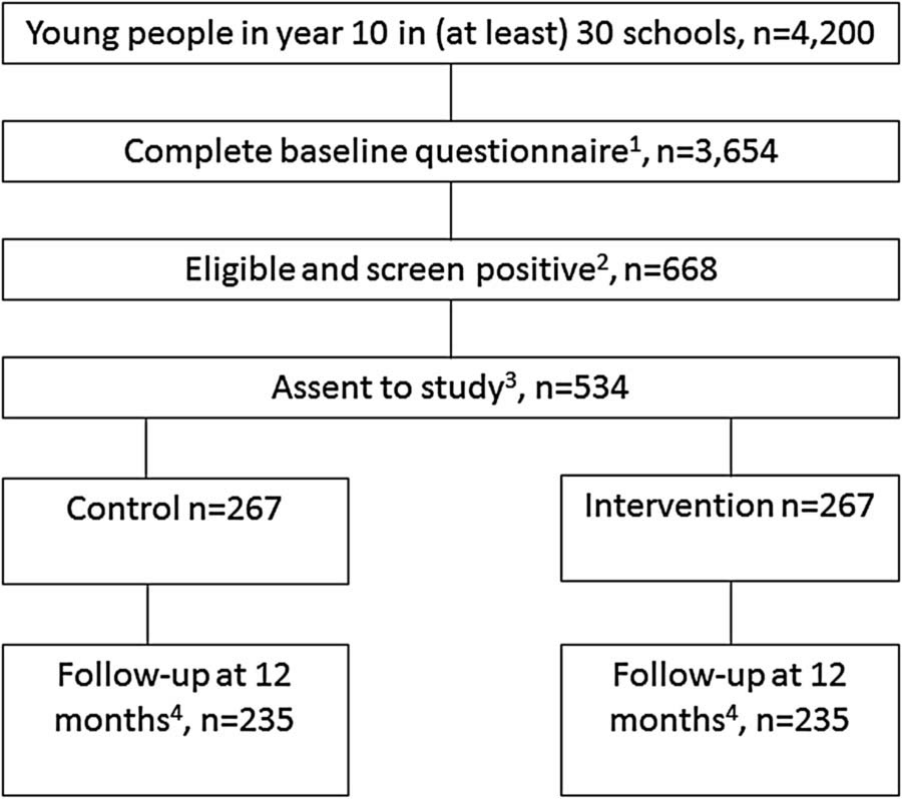
Study flowchart.

Using estimates from the pilot trial (mean year group size=210, 87% completing baseline questionnaire, 20% being positive on A-SAQ and leaving contact details, 80% recruited to trial and 88% providing data at 12 months follow-up), achieving follow-up data on 235 young people per arm the sample size has been calculated to have a 90% power to detect a standardised difference of 0.3 (which equates to a ratio of 1.5 in geometric means in total alcohol units in 28 days) using a significance level of 5% (figure 1).

### Analysis

#### Baseline data

Descriptive statistics (comparisons of percentages, means or medians as appropriate) will be used to report the pupil-level baseline data, and completeness of intervention received between those allocated to the two trial arms.

#### Primary outcome

The primary effectiveness analysis will be by intention-to-treat. Multiple linear regression will be used to compare the primary outcomes between the two randomisation groups at 12 months, adjusting for any imbalance in key covariates, including school.

#### Secondary outcomes

The secondary outcomes will be analysed in a similar manner. Comparisons of means will be presented as mean differences or ratios of geometric means (if a logarithmic transform is necessary for skewed data) with 95% CIs  ORs and 95% CIs will be presented for binary outcomes. Exploratory analyses will also be undertaken, for example, to examine differences in outcome by gender, deprivation and extent of intervention received, though there is limited power to investigate these comparisons. These moderators have been included so as to consider whether the ABI may need to be targeted when delivered, should it be shown to be effective. We will consider any difference in attrition rates, and any non-randomness of the attrition, when comparing outcomes between the two groups. The pattern of missing observations because of loss to follow-up will be examined to determine the extent of missingness, and whether it is missing at random or is informative. If data are missing to a sufficient extent, the use of appropriate multiple imputation techniques will be considered.

#### Health economics

The economic component will include a within trial cost-utility and cost-consequence analysis and, as described below, a model based analysis taking the perspective of the UK public sector (NHS, educational, social and criminal services). The cost-utility analysis will use measures of effectiveness limited to health-related quality of life as measured by EQ-5D Y. The cost-consequence analysis will take the same perspective for costs but will present these alongside all of the primary and secondary measures of effectiveness outlined above. The follow-up for the within trial analyses will be 12 months, so discounting will not be conducted. For the model-based analysis, the time horizon will be longer (potentially up to the participant's life time) and costs and effects will be discounted at 1.5%, the UK recommended rate for public health interventions,^35^ with a sensitivity analysis used to explore the impact of higher (and lower) discount rates.

#### Within-trial analysis: cost-utility and cost-consequence analyses

For each trial participant, the use of health, educational, criminal and social care services will be elicited using the S-SUQ administered at baseline (with a recall period of 3 months) and 12 months. Further cost data will come from the learning mentor time case diaries completed by the learning mentors for each contact. Costs for healthcare and social services will be obtained from standard sources such as NHS reference (http://www.gov.uk), the British National Formulary^36^ for medications, Unit costs of Health and Social Care^37^ for contacts with primary care. Further data will come from the study centres themselves. Data on the use of educational services will be elicited via the questionnaire. As part of the pilot trial, we confirmed with the expert group the type of services relevant to collect and have also added further questions related to days missed from school and truancy.

Learning mentor training costs will be included and will need to be apportioned according to scaled up practice. This will be informed by data from the training conducted as part of the trial and through expert opinion. The time of educational staff will be sought through a parallel costing exercise in which these staff will be asked to provide information on the impact of the intervention on their workload. With respect to learning mentors, a detailed proforma (case diary) was developed and tested in the pilot to capture resource use for cost-effectiveness analyses, and this new tool will be used in this study. With respect to school building and other large capital items, the opportunity cost will be considered. Some resources (eg, buildings) will exist with or without the intervention and the intervention may not displace any other activity. In this circumstance, the opportunity cost of the building would be zero. However, costs might be incurred in terms of heat, power and light, and these data will be captured using standard costing methods.^38^ For each participant, measures of use of resources will be combined with unit costs to provide a cost for that participant. We anticipate that the price year adopted for the base case analysis will be 2017 when the final analysis is conducted.

The EQ-5D Y will be administered at baseline and 12 months with UK population tariffs^39^ used. Health state utilities from the EQ-5D Y will then be used to estimate QALYs using the area under the curve approach.^26^

## Qualitative study

### Aims

In addition to the main trial, an embedded qualitative study will be conducted. The qualitative study will:

- Explore the delivery and efficacy of screening and brief intervention approaches in the school setting, and to elicit participants' experiences of the study;
- In interviews with school staff: explore the mechanisms and processes of implementing the SIPS JR-HIGH intervention to understand how this brief intervention could become embedded in the work role of school staff, the prioritisation of educational or well-being work, the scope for team or individual professional input, staff skill mix and turnover, resources, role development and training needs, and participants' assent;
- In interviews with young people: explore their experiences of taking part in the study and their views on any derived benefits, adverse events or improvements;
- In interviews with parents: explore their views on school-led interventions for adolescent alcohol use, issues relating to parental consent to take part in such interventions and the appropriateness of school-led health promotion work across the school-home interface.

### Sample

At each of the four research sites, we will seek to interview a minimum of: two teachers and four learning mentors from different schools (24 interviews in total); and participating young people from a random selection of included schools, with an even representation of males and females across both trial arms (40 interviews). We will also interview parents of young people in attendance at schools in each of the four sites (16 interviews in total). We will endeavour to include mothers and fathers (cohabiting and lone parents) in the sample as well as parents of boys and girls covering different cultural groups. Variation will also be sought in terms of drinking risk status of the young people (based on A-SAQ screening data) and socioeconomic status of young people and parents (as measured by index of multiple deprivation rank of school and the first part of the pupil's postcode at baseline and A-SAQ score at follow-up). Teachers and learning mentors will be sampled according to variation of socioeconomic status of the school where they are employed. Data saturation for either data set (school staff or young people) will be defined as no substantively new themes having emerged from the analysis of three consecutive interviews.^40^

### Recruitment, consent and assent

Research Coordinators from each of the four sites will disseminate an invitation letter and information leaflets to all participating teachers and learning mentors. In addition, school staff from each of the four sites will disseminate the letter and information leaflet to all young people. Schools will text parents and direct them to an online platform containing the invitation letter and information leaflet.

The online facility will offer the ability to opt-in to the study, which parents, young people and school staff can complete if they wish to participate in the qualitative interviews. Alternatively, they can contact the research coordinator to arrange a suitable interview date by email or telephone.

### Consent and assent

All participants will be given a copy of a relevant information sheet and school staff and parents will be asked to complete a consent form and young people an assent/consent form before taking part in the qualitative component of the study.

### Study design

Semistructured face-to-face interviews will be conducted with all participants. All interviews will be audio-recorded and transcribed verbatim.

### Analysis

Data from all interviews will be subjected to thematic analysis, which is appropriate for qualitative health research which seeks to explore key concepts pertinent to the research aims, but without presupposing a rigid framework and a priori selection of key themes.^41^ This analytic strategy is characterised by an inductive approach, in which analysis is open and flexible, allowing themes to be generated from the research, in order that findings have relevance to applied research questions, but are not led by the researchers, as would a deductive approach dictate.^42^ ^43^ Data will be coded by a qualitative researcher following standard thematic analysis procedures. That said, our development of the discussion guide for the interviews will be informed by theory on the likelihood of embedding study interventions in clinical practice, namely Normalization Process Theory.^44^ It is expected that the discussion guide will include questions linked to intervention implementation, such as role legitimacy (appropriateness of role/parental views, any role conflicts), adequacy (training, how the children are identified, how the intervention is conducted) and support (time available, support from school, parents). This theory considers factors that affect implementation in four key areas: how people make sense of a new practice (coherence); the willingness of people to sign-up and commit to the new practice (cognitive participation); their ability to take on the work required of the practice (collective action); and activity undertaken to monitor and review the practice (reflexive monitoring). The approach is increasingly used in studies of the implementation of interventions in healthcare (http://www.normalizationprocess.org). Data from interviews with young people and parents will also be analysed inductively first through open coding and thematic analysis, and will follow the principles of constant comparison thereafter.^45^ In this way, a qualitative researcher will read the interview transcripts and identify important or recurrent themes emerging from the transcripts. These emergent themes will be used to code the remaining transcripts, with open coding of any new themes that may emerge to expand on the emerging theory/results. In addition to NPT, we will also develop the discussion guide accounting for Bourdieu's concept of habitus;^46^ ^47^ an approach used successfully by this team in qualitative work with young people within the age range of this study^48^ and their parents.^49^ Habitus represents a set of tastes and dispositions shared with others in social space,^49^ providing cultural norms and historic precedents continually reproduced through practice.^50^ The use of this theoretical framework offers a mechanism in which to explore young people's socially constructed responses to brief intervention. Furthermore, the reciprocal idea of an individual and their interaction with society accords with the social learning underpinning of brief intervention.

At least one other qualitative expert will read and second-code a proportion of interviews with young people and staff to check for coding accuracy; divergence interpretations and enrich the analysis.^41^ Coded data will be reviewed to produce a detailed description of key results. We will use NVivo software to aid indexing and charting. Analysis will be ongoing throughout the process of data collection, and will be discussed at regular meetings within the research team in order to identify areas for closer consideration (including negative case analysis) and to enhance credibility of the analytical process and data interpretation.^51^ Qualitative analysis will take place prior to outcomes analysis, in keeping with published recommendations.^52^

### Triangulation

Once the qualitative interviews from this study have been carried out and analysed separately, they will be combined with the quantitative data from the main trial at the ‘analysis/interpretation’ phase, which is a process often described as ‘triangulation’.^53^ In our study, data will be reconciled by adopting a model which relies on the principle of complementarity.^54^ Within this approach it is explicitly recognised that qualitative and quantitative methods may be used to examine different aspects of an overall research question.^53^

### Study reporting and publications

If the intervention is shown to be effective and efficient we will develop a manual alcohol screening and brief intervention protocol to facilitate uptake/adoption in routine practice in secondary schools in England.

It is planned to publish this study in peer-reviewed articles and to present data at national and international meetings. Results of the study will also be reported to the sponsor and funder and will be available on their website. All manuscripts, abstracts or other modes of presentation will be reviewed by the trial steering group and funder prior to submission. Individuals will not be identifiable in any study report.

## Footnotes

i Cost-effectiveness will be established to determine whether it is worthwhile to roll-out the ABI across schools in England, should it be found to be effective.
