# Supplementary material for: Multicentre individual randomised controlled trial of screening and brief alcohol intervention to prevent risky drinking in young people aged 14–15 in a high school setting (SIPS JR-HIGH): study protocol
Source: BMJ Open. 2016 Dec 23;6(12):e012474. doi: 10.1136/bmjopen-2016-012474 (PMC5223663; doi:10.1136/bmjopen-2016-012474)
Supplement: supplementary appendix [file bmjopen-2016-012474supp_appendix1.pdf]

APPENDIX 1

A multi-centre individual-randomised controlled trial of screening and brief alcohol intervention to prevent risky drinking in young people aged 14-15 in a high school setting (SIPS JR-HIGH): **Study protocol**

Full Title: A multi-centre individual-randomised controlled trial of screening and brief alcohol intervention to prevent risky drinking in young people aged 14-15 in a high school setting (SIPS JR-HIGH)

Short title: SIPS JR-HIGH

Chief Investigator: Professor Dorothy Newbury-Birch[1]
Co-applicants: Professor Eileen Kaner[2], Professor Elaine McColl[2,7], Denise Howel[2], Elaine Stamp[2], Dr Ruth McGovern[2], Dr Stephanie Scott[2], Professor Luke Vale[2], Professor Liz Todd[2], Professor Simon Coulton[3], Professor Colin Drummond[4], Dr Paolo Deluca[4], Professor Harry Sumnall[5], Les Tate[6]
Principal Investigators (sites): Professor Dorothy Newbury-Birch (North East), Professor Harry Sumnall (North West), Professor Simon Coulton (Kent), Professor Colin Drummond/Dr Paolo Deluca (London).
Project Manager: Dr Emma Giles[1]
Senior Trial Manager (NCTU): Claire Macdonald[7]
Research Co-ordinators: Dr Grant McGeechan[1], Jayne Ogilvie[3], Dr Sadie Boniface[4], Jo Frankham[5], Grant Stanley[5]
Qualitative Researcher: Dr Grant McGeechan[1]
Database Manager: Nicola Howe[2]
Administrative Assistant: Robert Sayer[1]

[1]Health and Social Care Institute, Teesside University, [2]Institute of Health and Society, Newcastle University, [3]University of Kent, [4]Kings College London, [5]Liverpool John Moores University, [6]North Tyneside Council, [7]Newcastle Clinical Trials Unit, Newcastle University

- **ISRCTN Number:** ISRCTN45691494
- **Ethics approval number:** No 164/15
- **Protocol Version and Date:** Version 1.4. 26.04.2015
- **Funded by / Grant reference:** NIHR PHR 13/117/02
- **Sponsored by:** Newcastle University

1. Protocol contacts

Chief Investigator: Professor Dorothy Newbury-Birch, Professor of Alcohol and Public Health Research, Health and Social Care Institute, Teesside University, Middlesbrough. TS1 3BA. D.newbury-birch@tees.ac.uk

Project Manager: Dr Emma Giles, Senior Research Lecturer in Public Health, Health and Social Care Institute, Teesside University, Middlesbrough. TS1 3BA. E.giles@tees.ac.uk

Newcastle Lead: Professor Eileen Kaner, Institute Director, Newcastle University, Institute of Health and Society, Newcastle upon Tyne, Tyne and Wear, NE2 4AX. Eileen.kaner@newcastle.ac.uk

Sponsor: Mrs Lois Neal, Faculty of Medical Sciences, Newcastle University, Framlington Place, Newcastle upon Tyne, Tyne and Wear, NE2 4HH. lois.neal@newcastle.ac.uk

Senior Research Interventionist: Dr Ruth McGovern, Newcastle University, Institute of Health and Society, Newcastle upon Tyne, Tyne and Wear, NE2 4AX. r.mcgovern@ncl.ac.uk

Statistics: Denise Howel, Senior Lecturer in Epidemiological Statistics, Newcastle University, Institute of Health and Society, Newcastle upon Tyne, Tyne and Wear, NE2 4AX. denise.howel@newcastle.ac.uk

QOL/Health Economics: Professor Luke Vale, Newcastle University, Health Foundation Chair in Health Economics. Newcastle University, Institute of Health and Society, Newcastle upon Tyne, Tyne and Wear, NE2 4AX. luke.vale@newcastle.ac.uk

Senior Trial Manager (NCTU): Claire Macdonald, Newcastle University, Newcastle Clinical Trials Unit, 1-4 Claremont Terrace, Newcastle upon Tyne, Tyne and Wear, NE2 4AE. claire.macdonald@ncl.ac.uk

Database Manager: Nicola Howe, Newcastle University, Newcastle Clinical Trials Unit, 1-4 Claremont Terrace, Newcastle upon Tyne, Tyne and Wear, NE2 4AE. nicola.howe@newcastle.ac.uk

Administrative Assistant: Robert Sayer, Health and Social Care Institute, Teesside University, Middlesbrough. TS1 3BA. R.sayer@tees.ac.uk

Emergency contact (e.g. out of office hours): Professor Dorothy Newbury-Birch 07980629456

2. Protocol signature page

REPRESENTATIVE OF THE RESEARCH SPONSOR

**Name:** Lois Neal	**Position:** Assistant Registrar
**Signature:**	**Date:**

CHIEF INVESTIGATOR

**Name:** Professor Dorothy Newbury-Birch	**Position:** Professor of Alcohol and Public Health Research
**Signature:**	**Date:**

PROJECT MANAGER

**Name:** Dr Emma L Giles	**Position:** Senior Research Lecturer in Public Health
**Signature:**	**Date:**

STATISTICIAN

**Name:** Ms Denise Howel	**Position:** Senior Lecturer in Epidemiological Statistics
**Signature:**	**Date:**

HEALTH ECONOMIST

**Name:** Professor Luke Vale	**Position:** Health Foundation Chair in Health Economics
**Signature:**	**Date:**

2.1 Principal Investigator signature

I confirm that I have read and understood protocol 1.4 dated 26.04.2015. I agree to comply with the study protocol, the principles of Good Clinical Practice (GCP), research governance, clinical trial regulations and appropriate reporting requirements.

Print Name
Site Name/I.D.

Signature ……………………………… Date ………..

Contents

1. Protocol contacts 2
2. Protocol signature page 3
2.1 Principal Investigator signature 4
3. Glossary of abbreviations 7
4. Responsibilities 8
4.1 Trial management 8
4.2 Trial conduct at sites 8
4.3 The Caldicott principles 9
5. Protocol summary 11
6. Background 12
6.1 Consequences of drinking in early life 12
6.2 Primary and secondary preventative interventions for risky drinking 12
6.3 Brief intervention 13
6.4 Rationale for current study 13
7. Research aim and objectives 14
7.1 Primary objective 14
7.2 Secondary objectives 14
8. Study design 15
9. Outcome trial assessments 15
9.1 Baseline assessments 15
9.2 12 month assessments 15
9.3 Primary outcome measure: 16
9.3.1 Secondary (effectiveness) outcomes measures: 16
9.3.2 Secondary (health economic) outcome measures: 16
9.4 Definition of end of study: 16
10. Participants 16
10.1 Socioeconomic context and inequalities 16
10.2 Inclusion criteria 17
10.3 Exclusion criteria 17
11. Trial procedures 17
11.1 Training 17
11.2 Control arm 18
11.3 Intervention 18
12. Randomisation 18
12.1 Questionnaire and intervention process 18
12.2 Flowchart of study 21
13. Screening, recruitment and assent 21
13.1 Screening and eligibility criteria 21
13.2 Option to opt-out of screening 21
13.3 Screening for the trial 21
13.4 Data collection 22
13.4.1 Baseline data collection 22

13.4.2 Invitation to meet with learning mentors 22
13.5 Assent procedures 23
13.4.3 12-month follow-up 23
13.4.4 Intervention fidelity 24
14. Project timetables and milestones 24
15. Statistical considerations 24
15.1 Sample size calculation 24
15.2 Analysis 25
15.2.1 Baseline data 25
15.2.2 Primary outcome 25
15.2.3 Secondary outcomes 25
15.2.4 Interim analyses 25
15.2.5 Missing data 25
16. Health economics 25
16.1 Within trial analysis cost-utility and cost-consequence analyses 26
16.2 Model based analysis 27
17. Qualitative work 27
18. Triangulation 27
19. Compliance and withdrawal 28
19.1 Assessment of compliance 28
19.2 Withdrawal of participants 28
20. Data monitoring, quality control and quality assurance 28
21. Adverse event monitoring and reporting 29
22. Ethics and regulatory issues 29
23. Research governance 29
24. Confidentiality 30
25. Insurance and finance 30
26. Study report/publications 31
27. References 31

3. Glossary of abbreviations

AUDIT	Alcohol Use Disorders Identification Test
A-SAQ	Student Alcohol Questionnaire
BECCI	Behaviour Change Counselling Index
C.I.	Confidence Intervals
C-RCT	Cluster Randomised Control Trial
DMES	Data Monitoring and Ethics Committee
DMQ	Drinking Motives Questionnaire
EQ-5D-Y	European Quality of Life Five Dimension – Youth
FRAMES	Feedback, Responsibility, Advice, Menu, Empathy and Self-efficacy
GCP	Good Clinical Practice
MRC	Medical Research Council
NHS	National Health Service
NHS EED	National Health Service Economic Evaluation Database
NICE	The National Institute for Health and Care Excellence
NIHR PHR	National Institute of Health Research, Public Health Research
PI	Principal Investigator
PSHE	Personal Social and Health Education Lessons
QALYS	Quality Adjusted Life Years
RAPI	Rutgers Alcohol Problems Inventory
RCT	Randomised Control Trial
S-SUQ	Short Service Use Questionnaire
TLFB	Time Line Follow Back
TOC	Trial Oversite Committee
WEMWBS	Warwick Edinburgh Mental Well-Being Scale

4. Responsibilities

Sponsor: Newcastle University are the award holders and will act as the sponsor for this study.

Funder: NIHR PHR is funding this study.

Trial management: A Trial Management Group (TMG) will be appointed and will be responsible for overseeing the progress of the trial. The day-to-day management of the trial will be co-ordinated by the Project Manager. A Trial Steering Group (TSC) and a separate Data Management and Ethics Committee (DMEC) will also be appointed to monitor trial data.

Chief Investigator: The Chief Investigator will have overall responsibility for the trial.

Principal Investigators: The Principal Investigators (PIs) will have overall responsibility for the conduct of the study at a particular trial site.

4.1 Trial management

The following functions falling under the responsibility of the sponsor will be delegated to Professor Dorothy Newbury-Birch [Chief Investigator]:

- Ethics Committee Opinion (including application for research ethics committee favourable opinion, notification of protocol amendments and end of trial, site specific assessment and local approval).
- Good Clinical Practice and Trial Conduct (including GCP arrangements, data monitoring, emergency and safety procedures).

Administration of funding for the study will be carried out by Newcastle University who hold the award. Professor Eileen Kaner is the lead for Newcastle University.

4.2 Trial conduct at sites

Site PI responsibilities

- Study conduct and the welfare of study subjects.
- Familiarity with the study conditions.
- Compliance with the protocol, documentation of any protocol deviations and reporting of all serious adverse events.
- Screening and recruitment of subjects.
- Compliance with the Principles of GCP, the Research Governance Framework for Health and Social Care, the Data Protection Act and any other relevant legislation and regulatory guidance.
- Ensuring that no participant is recruited into the study until all relevant regulatory permissions and approvals have been obtained.
- Obtaining written informed assent from participants prior to any study specific procedures.
- The PIs shall be qualified by education, training and experience to assume responsibility for the proper conduct of the trial. S/he shall provide a current signed and dated curriculum vitae as evidence for the Trial Master File.
- Ensuring Study Site team members are appropriately qualified by education, training and experience to undertake the conduct of the study.
- Availability for TSCs, DMECs, monitoring visits and in the case of an audit.

- Maintaining study documentation and compliance with reporting requests.
- Maintaining a site file, including copies of study approval, list of subjects and their signed informed assent forms.
- Documenting appropriate delegation of tasks to other study personnel e.g. Research Co-ordinators.
- Ensuring data collected is accurate, timely and complete.
- Providing updates on the progress of the trial.
- Ensuring subject confidentiality is maintained during the project and archival period.
- Ensuring archival of study documentation for a minimum of ten years following the end of the study, unless local arrangements require a longer period.

4.3 The Caldicott principles

Principle 1. Justify the purpose(s) for using confidential information: Every proposed use or transfer of personal confidential data within or from an organisation should be clearly defined, scrutinised and documented, with continuing uses regularly reviewed, by an appropriate guardian.

How we will abide by Principle 1: Should we need to transfer personal data between Newcastle and Teesside Universities we will keep a log of the transfer, who requested and who executed the transfer, together with the reason for the transfer. This log will be kept on a password protected Excel file.

Principle 2. Don't use personal confidential data unless it is absolutely necessary: Personal confidential data items should not be included unless it is essential for the specified purpose(s) of that flow. The need for patients to be identified should be considered at each stage of satisfying the purpose(s).

How we will abide by Principle 2: We will gather limited personal data, including name and first part of postcode for trial participants. This is to allow us to map behaviours to socio demographic characteristics.

Principle 3. Use the minimum necessary personal confidential data: Where use of personal confidential data is considered to be essential, the inclusion of each individual item of data should be considered and justified so that the minimum amount of personal confidential data is transferred or accessible as is necessary for a given function to be carried out.

How we will abide by Principle 3: We will ask for name and class only to minimise the amount of personal we collect from the young people. For trial participants we will ask for the first part of their postcode.

Principle 4. Access to personal confidential data should be on a strict need-to-know basis: Only those individuals who need access to personal confidential data should have access to it, and they should only have access to the data items that they need to see. This may mean introducing access controls or splitting data flows where one data flow is used for several purposes.

How we will abide by Principle 4: Only the Study Research Administrator at Teesside University will have access to all of the information to ensure allocation concealment in the trial. The data will be accessed on a need-to-know basis only.

Principle 5. Everyone with access to personal confidential data should be aware of their responsibilities: Action should be taken to ensure that those handling personal confidential data - both clinical and non-clinical staff - are made fully aware of their responsibilities and obligations to respect patient confidentiality.

How we will abide by Principle 5: We will be providing training to all active researchers in the trial to ensure they understand confidentiality principles.

Principle 6. Comply with the law: Every use of personal confidential data must be lawful. Someone in each organisation handling personal confidential data should be responsible for ensuring that the organisation complies with legal requirements.

How we will abide by Principle 6: The research sponsor will ensure that all use of personal data will be lawful.

Principle 7. The duty to share information can be as important as the duty to protect patient confidentiality: Health and social care professionals should have the confidence to share information in the best interests of their patients within the framework set out by these principles. They should be supported by the policies of their employers, regulators and professional bodies.

How we will abide by Principle 7: We will abide by the policies of participating organisations.

5. Protocol summary

**Trial Title**	A multi-centre individual-randomised controlled trial of screening and brief alcohol intervention to prevent risky drinking in young people aged 14-15 in a high school setting (SIPS JR-HIGH)
**Acronym (short title)**	SIPS JR-HIGH
**Protocol version and date**	1.4 26.04.2016
**Summary of Trial Design**	A four-centre, individually randomised two armed Randomised Controlled Trial (RCT) incorporating a control arm of usual practice on alcohol issues and a 30 minute brief intervention arm.
**Summary of Participant Population**	Young people aged 14-15 years inclusive, whose parents do not opt them out of the study, scoring positively on the A-SAQ, leave their name and willing and able to provide informed assent for intervention and follow-up.
**Planned Sample Size**	4,200 pupils in year 10; with 235 in each arm at the 12-month follow-up.
**Planned Number of Sites**	At least five schools in each of the four geographical sites: North East, North West, London and Kent.
**Study Intervention**	30 minute brief alcohol intervention.
**Follow Up Duration**	At 12-months post intervention; completion of a questionnaire.
**Planned Trial Period**	01 September 2015 - 31 December 2017.

Primary objective: Total alcohol consumed in the last 28 days.
Outcome measure: Time Line Follow-Back (TLFB) questionnaire at 12-month follow-up.
Secondary (effectiveness) objective: To measure % days abstinence over last 28 days; risky drinking; smoking behaviour; alcohol-related problems; drunkenness during the last 30 days; and emotional wellbeing.
Outcome measures: Drinks per day and days>2 units from 28 day TLFB; risky drinking using the Student Alcohol Questionnaire (A-SAQ), Alcohol Use Disorders Identification Test (AUDIT) and TLFB; smoking behaviour and alcohol related problems using the Rutgers Alcohol Problems Inventory (RAPI); drunkenness dichotomised as ‘never’ or ‘once or more’; emotional wellbeing using the Warwick Edinburgh Mental Health Well-being Scale (WEMWBS) and Drinking Motives Questionnaire (DMQ).
Secondary (health economics) objectives: To measure Quality of Life Years (QALY) and health state utility and cost-consequences at 12 months.
Outcome measures: Quality of life and health state utility measured using the European Quality of Life Five Dimension (EQ-5D Y) [1]; QALYs estimated using general population tariffs from responses to EQ-5D Y administered and scored at baseline and 12 months; National

Health Service (NHS), educational, social, and criminal services data estimated using a modified Short Service Use Questionnaire (S-SUQ) and a learning mentor diary sheet developed in the pilot study; Incremental cost per QALY gained at 12 months; Cost-consequences presented in the form of a balance sheet for outcomes at 12 months; depending on findings, modelled estimates of incremental cost per QALY and cost-consequences in the longer term.

6. Background

Adolescents in England are amongst the heaviest drinkers in Europe [1]. The percentage of young people who have ever had an alcoholic drink in England increases with age from 6% of 11 year olds to 72% of 15 year olds, and the prevalence of drinking in the last month rises from 2% of 11 year olds to 43% of 15 year olds [2]. Whilst drinking typically increases over adolescence, there has been a reduction over time across all ages of adolescents, although amongst those who drink they typically consume a higher volume. Nevertheless, drinking can have adverse impacts on health and social (including learning) outcomes for the many young people who are drinking alcohol.

6.1 Consequences of drinking in early life

The impact of alcohol on the development and behaviour of young people has been well researched in early [3], middle [4] and late adolescence [5]. It is now well known that young people are much more vulnerable than adults to the adverse effects of alcohol, due to a range of physical and psycho-social factors which often interact [6]. These adverse effects include: physiological factors [4]; neurological factors due to changes that occur in the developing adolescent brain after alcohol exposure [7]; cognitive factors due to psychoactive effects of alcohol which impair judgement and increase the likelihood of accidents and trauma [8]; and social factors which arise from a typically high-intensity drinking pattern (often called 'binge drinking') which leads to intoxication and risk-taking behaviour [9].

Our definition of risky drinking encompasses commonly understood concepts of hazardous drinking (at a level or pattern that increases the risk of physical or psychological problems), harmful drinking (defined by the presence of these problems) and binge drinking (risky single occasion high intensity drinking which can be episodic) as well as the Department of Health concepts of increasing and high risk drinking [10]. Evidence suggests that risky drinking among young people occurs commonly in the context of other forms of challenging behaviour such as aggression and risk-taking [11]. The Chief Medical Officer for England has provided recommendations on alcohol consumption in young people [12] based on an evidence review of the risks and harms of alcohol to young people [6]. The recommendations state that children should abstain from alcohol before the age of 15 and those aged 15-17 are advised not to drink, but if they do drink it should be no more 3-4 units and 2-3 units per week in males and females respectively, on no more than one day per week [12] which equates to adult daily drinking recommendations.

6.2 Primary and secondary preventative interventions for risky drinking

There is a large volume of evidence on primary prevention to reduce risky drinking in the school setting [13, 14]. Such prevention is directed at all young people, whether they drink alcohol or not, and aims to delay the age that drinking begins, often via general health education. This body of work has shown mixed results with only a small number of programmes reporting positive outcomes [14] and this body of work has been reported to be

methodologically weak [15]. Secondary prevention, i.e. targeting interventions at young people who are already drinking alcohol, may be a more effective and efficient strategy since the intervention will have more salience for the individuals receiving it.

This secondary prevention generally consists of screening (to identify relevant recipients) followed by structured advice or counselling of short duration which is aimed at reducing alcohol consumption or decreasing problems associated with drinking [16]. The interventions are based on social cognitive theory (from health psychology) which is drawn from social learning theory [17]; these theories regard behaviour to be the result of an interaction between individual, behavioural and environmental factors. It is assumed that each individual has cognitive (thinking) and affective (feeling) attributes that affect not only how they behave but also how their behaviour is influenced and/or reinforced by aspects of the external world. Thus, brief interventions generally focus on individuals' beliefs and attitudes about behaviour, their sense of personal confidence (self-efficacy) about changing beliefs and attitudes and a focus on how an individual's behaviour sits in relation to other people's actions (normative comparison).

6.3 Brief intervention

A key feature of brief intervention is that it is designed to be delivered by generalist practitioners (not addiction specialists) and is targeted at individuals who may not be aware they are experiencing alcohol-related risk or harm. The goal is usually reduced alcohol consumption or a decrease in alcohol-related problems [18]. There is variation in the duration and frequency of brief intervention [19] but there are two broad types: simple structured advice - based on the FRAMES model (feedback, responsibility, advice, menu, empathy and self-efficacy) and motivational interviewing [20]. Since the time available for delivering brief intervention may not allow for motivational interviewing in its full form [19], its ethos and techniques have been distilled into a more directive format called Behaviour Change Counselling [21] which has been successfully used in a number of UK trials [22-25]. Existing evidence described above demonstrates that alcohol screening and brief intervention for young people have been successful for selected individuals, in certain settings.

6.4 Rationale for current study

The Chief Medical Officer for England has stated that school is seen as a key resource in the prevention, detection and treatment for risky drinking [26]. However, the current evidence is limited as it relates primarily to white, USA-based study participants and provides insufficient evidence to be confident about the use of alcohol screening and brief intervention to reduce excessive drinking and/or alcohol-related harm in younger adolescents aged under 16 and in a school setting in the UK [27-29]. Nevertheless, there is evidence that the most practical and effective forms of brief intervention in this setting are those based upon the FRAMES model. Specifically approaches containing personalised feedback about a young person's drinking behaviour with motivational interviewing approaches, such as behaviour change counselling, can help to reduce levels of alcohol-related risk [23].

This current work builds on the evidence base by focusing on screening and brief intervention to reduce risky drinking in younger adolescents (aged 14-15). The proposed study follows on from the SIPS JR-HIGH pilot feasibility study which was funded under the National Institute of Health Research Public Health Programme (NIHR PHR) commissioned call 10/3002 Alcohol and Young People: Interventions to prevent risky drinking of alcohol by school aged children

and young people [30]. The trial was registered on the ISRCTN register as ISRCTN07073105. The pilot feasibility trial was a three-arm cluster-randomised control trial (c-RCT) (with randomisation at the school level) with an integrated qualitative component to assess the feasibility and acceptability of a future definitive trial of brief alcohol intervention in a school setting. The trial measured recruitment and retention to the study, and explored facilitators and barriers to the use of these interventions with year 10 pupils (aged 14-15) in seven schools in the North East of England [23].

In our pilot feasibility study, young people who screened positively on a single alcohol screening question (collected in the context of a baseline classroom survey of drinking and other health behaviours) and assented to take part in our trial were randomised to either: provision of an advice leaflet (control arm, n=two schools); a 30-minute brief interactive session which combined structured advice and behaviour change counselling techniques delivered by the school learning mentor, and an advice leaflet (Intervention 1, n=two schools); or the 30-minute brief interactive session and an advice leaflet with the addition of a 60-minute session involving family members delivered by the school learning mentor (Intervention 2, n=three schools). Trial participants were followed-up at 12 months (88% retention). The results showed that it was not possible to carry out the second arm of the trial with parents, therefore the definitive study will only include two arms. As there are only two arms to the trial it is feasible to change to an individually randomised trial.

7. Research aim and objectives

The aim of the study is to evaluate the effectiveness and cost-effectiveness of alcohol screening and brief intervention to reduce risky drinking in young people aged 14-15 in the English high school setting.

7.1 Primary objective

To conduct an individually randomised controlled trial to evaluate the effectiveness and cost-effectiveness of alcohol screening and brief intervention for risky drinkers compared to standard usual practice on alcohol issues conducted by learning mentors with young people aged 14-15 in the school setting in the North East, North West, South East and London, England. Effectiveness is measured by total alcohol consumed in the last 28 days as measured by the 28 day TLFB.

7.2 Secondary objectives

- To measure effectiveness in terms of % days abstinence over last 28 days; risky drinking; smoking behaviour; alcohol-related problems; drunkenness during the last 30 days; and emotional wellbeing.
- To measure the cost-effectiveness of the intervention in terms of quality of life and health state utility; QALYs; Service use costs and cost-consequences at 12 months post intervention.
- To monitor the fidelity of alcohol screening and brief intervention delivered by learning mentors in the school setting.
- To explore barriers and facilitators of implementation with staff.
- To explore young people’s experiences of the intervention and its impact upon their alcohol use.

- If the intervention is shown to be effective and efficient to: develop a manualised screening and brief intervention protocol to facilitate uptake/adoption in routine practice in secondary schools in England.

8. Study design

This is a multicentre individually randomised controlled trial comparing effectiveness and cost-effectiveness of alcohol brief intervention with treatment as usual in young people aged 14-15 in the school setting who screen positive for risky drinking using the A-SAQ.

9. Outcome trial assessments

9.1 Baseline assessments

Baseline data will be collected though self-completion questionnaires.

- Age of first smoking and how many cigarettes were smoked in the past 30 days [1].
- Alcohol use frequency, quantity (on a typical occasion) and binge drinking assessed using the modified 10 question AUDIT [31] which has been shown to be a highly sensitive tool for college students [32].
- Alcohol related problems assessed using the validated RAPI which includes measures on aggression [33].
- Drinking motives assessed using the 20-item DMQ. This tool uses a six-point Likert scale, which measures motives to drinking across four domains (social, coping, enhancement and conformity). Higher scores within each domain indicate stronger endorsement of positive reinforcement received through consumption of alcohol [34].
- Use of NHS, educational, social, and criminal services data elicited using a modified S-SUQ to capture health and social service use costs [35].
- The 14 item WEMWBS to assess general psychological health [36].This tool uses a five-point Likert scale which gives a score of one to five per question giving a minimum score of 14 and maximum score of 70. A higher WEMWBS score indicates a higher level of mental well-being [37]. The EQ-5D Y, a valid measure for those aged 12 or older, will be used to measure health related quality of life [38]. Response will be converted into utility scores using the UK population algorithm.
- Two questions relating to sexual risk taking are included in the questionnaire. These are the same questions as in the pilot study [23]. These questions are: “After drinking alcohol, have you engaged in sexual intercourse that you regretted the next day?” and “After drinking alcohol, have you ever engaged in sexual intercourse without a condom?” Both questions can be answered with one of the three following options: I have never engaged in sexual intercourse, Yes, or No.
- Energy drink consumption will be assessed by asking young people how many times a week they drink energy drinks. Young people can answer never, less than once a week, 2-4 days a week, 5-6 days a week, every day once a day, and every data more than once a day.
- Demographic information will be collected: gender, ethnicity. The first part of the postcode will be collected for trial participants.

9.2 12 month assessments

All tools used at baseline (self-completion questionnaires) as well as the 28 day TLFB questionnaire (administered by a Research Co-ordinator).

9.3 Primary outcome measure:

Total alcohol consumed in the last 28 days, using the 28 day TLFB questionnaire [39] at 12-month follow-up.

9.3.1 Secondary (effectiveness) outcomes measures:

- % days abstinence over last 28 days, drinks per drinking day and days>2 units from 28 day TLFB;
- Risky drinking using the A-SAQ, AUDIT [31] and 28 day TLFB [39];
- Use of energy drinks;
- Smoking behaviour;
- Alcohol related problems using the RAPI [33] and sexual risk taking;
- Drunkenness during the last 30 days, dichotomised as 'never' and once or more [40];
- Emotional wellbeing using the WEMWBS [36] and drinking motives using the DMQ [34].

9.3.2 Secondary (health economic) outcome measures:

- Quality of life and health state utility measured using the EQ-5D Y [38];
- QALYs estimated using general population tariffs from responses to EQ-5D Y administered and scored at baseline and 12 months;
- NHS, educational, social, and criminal services data estimated using a modified S-SUQ and a learning mentor case diary developed in the pilot study;
- Incremental cost per QALY gained at 12 months;
- Cost-consequences presented in the form of a balance sheet for outcomes at 12 months;
- Depending on findings, modelled estimates of incremental cost per QALY and cost-consequences in the longer term.

9.4 Definition of end of study:

The end of study will be the last participant's final study contact, at 12 months follow up (trial end date of 31/12/2017).

10. Participants

Young people aged 14-15 in Year 10 in at least 30 Secondary/High schools/Academies in four centres: the North East of England, North West of England, Kent and London. Schools will be included if they have learning mentors (or equivalent members of pastoral staff including teachers fulfilling this role) employed by the school. Screening will take place in the personal, social and health education (PSHE) or equivalent lesson or registration class on a classroom by classroom basis. Interventions will take place in the learning mentor's classroom or office space.

10.1 Socioeconomic context and inequalities

In 2008, a survey of 1,250 young people living in deprived communities in Britain found that over a third did not know what a unit of alcohol was and did not understand the term binge drinking [41]. Of these young people, 39% drank up to 20 units per week and 15% drank over 20 units per week [41]. Thus the adverse effects of social deprivation on young people may be compounded by possible health and social problems related to heavy drinking. Usually, the alcohol harm paradox is primarily known within an adult context and this may be due to the

fact that generally average consumption is reported. It seems reasonable to extrapolate the phenomenon to young people and they may also experience adverse consequences due to parental effects. The proposed project will be working with schools in four geographical sites, which will provide a range of social strata. Individuals with lower socio-economic status generally experience disproportionately more alcohol-related problems than higher socio-economic status people (an outcome that is not always linked to drinking level) and so any reduction in consumption or concomitant problems that occurs as a result of our intervention is likely to benefit the lower socio-economic status group most. Recent data shows that uptake of free school meals (rather than rates of eligibility) is highest in inner London (69%) compared to the North East (57%), the North West (53%) and the South East (36%) [42]. We are also collecting individual postcode data (first part of postcode) for trial participants which will enable us to calculate Indices of Multiple Deprivation. Fourteen percent of the population in England and Wales are from a minority ethnic group. There are differences in ethnicity in our four proposed geographical sites. The non-white British population in the areas is: North East (5%); North West (10%); South East (9%) and London (40%) [43].

Inclusion and exclusion criteria have been chosen to maintain a balance between ensuring the sample is representative of the wider population whilst ensuring that the trial population are able to engage both with the intervention and follow-up.

10.2 Inclusion criteria

Young people aged 14-15 years inclusive, whose parents do not opt them out of the study, scoring positively on the A-SAQ, leave their name, and are willing and able to provide informed written assent for intervention and follow-up.

10.3 Exclusion criteria

- Already seeking or receiving help for an alcohol use disorder.
- Those with a recognised mental health or challenging behaviour.

11. Trial procedures

This study has been designed in line with the Medical Research Council (MRC) recommendations for evaluation of complex interventions and the pilot feasibility study has informed the development of this proposed study [23]. This proposal represents stage five of the MRC framework ‘evaluating a complex intervention’ and comprises a RCT with effectiveness, cost-effectiveness and qualitative elements [44]. The trial will incorporate individual randomisation of pupils within schools. The pilot feasibility study found the data collection tools easy to use for the young people involved with very low levels of non-completion. The primary outcome of alcohol consumption using the TLFB will only be measured at 12 months post intervention so as not to bias the control group’s responses.

Learning mentors (or equivalent members of pastoral staff employed by schools) will deliver the intervention. Local areas vary in their essential qualifications for appointment for learning mentors; however, as a minimum they need to have a good standard of general education, especially literacy and numeracy, as well as experience of working with young people.

11.1 Training

All learning mentors will receive school-based training in the study procedures and intervention. Some schools will have one learning mentor whilst other schools will have more.

Research Co-ordinators will work with the individual schools to reach a pragmatic solution to how many are trained for the trial. Learning mentors will be brought together at one of the schools in each geographical site for this training or carried out in individual schools. Such outreach training was found to be the most cost-effective implementation strategy for alcohol screening and brief intervention delivery in the pilot [23] and other settings [45]. Training for learning mentors will be carried out by the trained Research Co-ordinators using a simulated scenario within a training package developed and employed in the pilot. Simulated scenarios will be videotaped and learning mentors will be assessed by the trainer prior to embarking on the study with more support if needed. Learning mentors will be provided with support materials and on-going support and supervision will be provided by research staff working on, and in collaboration with, the project. Support on implementing screening and paperwork relevant to the research will be provided by the research team, with a Research Co-ordinator in each geographical site. Research staff and trainers will maintain regular contact with schools throughout the study period, including site visits and telephone and email support.

11.2 Control arm

Usual practice on alcohol issues as delivered normally to all students in PSHE lessons and curriculum delivered by class teachers. Young people will also be given a healthy lifestyle information leaflet with local sources of help with healthy lifestyle issues, by the learning mentor, to those that assent to the trial. Usual practice may vary from school to school and information related to this will be captured by researchers at both time points of the study.

11.3 Intervention

In addition to input equivalent to the control arm, the young people who are eligible and assent to participate will take part in a single 30-minute personalised interactive worksheet-based session which was developed during the pilot feasibility trial. This will be delivered by the learning mentor and will contain structured feedback about the individual student’s drinking behaviour, and advice about the health and social consequences of continued risky alcohol consumption. The intervention encompasses the elements of the FRAMES approach for eliciting behaviour change [20].

12. Randomisation

Young people will not know which arm they are randomised to when they agree to take part in the study, and nor will the learning mentor until they open the envelope. Pupils will be randomised in a 1:1 ratio to the intervention and control arms, with individual randomisation.

A statistician not otherwise involved with the study will produce a computer-generated allocation list using random permuted blocks to ensure allocation concealment. The statistician will be provided with a list of screening identification numbers (identifying the site, school and young person) for eligible participants, in the form of an Excel spreadsheet. Randomisation will be undertaken by this statistician and an updated spreadsheet, including allocation of the study arm, will be returned to the administrative assistant at Teesside University.

12.1 Questionnaire and intervention process

- Questionnaires are printed (n=4200). Then a screening number is attached to each questionnaire using a sticky label or automated printing.

- The screening number will identify the geographical site, the school, and the participant number. For example: NEF0001 [eg North East, FerryMoor School, participant number 0001/4200].
- Labelled questionnaires are inserted into envelopes at Teesside University by the allocated Research Associate (Jennifer Birch: JB) and the Alcohol and Public Health team at Teesside University.
- Questionnaires are batched by school by JB at Teesside University and couriered to geographical research sites.
- Research Co-ordinators take their 1,050 questionnaires to relevant individual schools.
- Year 10 pupils confidentially complete their questionnaires in school time[1]. They put the questionnaires into the spare blank envelope and seal.
- Research Co-ordinators collect the sealed envelopes from each school.
- Research Co-ordinators take the sealed envelopes back to respective Universities. They open the envelopes and create a pile of questionnaires which score positive on ASAQ **and** where young people have left their name (i.e. the young person is eligible). These are double checked.
- Research Co-ordinators create an excel spreadsheet of all those scoring positively who have left their name. They have one column for the screening number and one column for the name. They send this encrypted excel spreadsheet to JB at Teesside (TeamAlpha@tees.ac.uk).
- Over the next few months Research Co-ordinators then input questionnaire data into a secure validated clinical data management system (Elsevier's MACRO). Recording of A-SAQ and screening number of the potentially eligible takes priority and should be completed within a week of getting them from the schools. Inputting of all questionnaires (positive and negative) can occur within the year, with priority given to positive (with no name) questionnaires.
- The page from the questionnaire that has the young person's name and screening code on will be removed and will be couriered separately from the completed questionnaires back to Teesside University once entered into the MACRO system.
- JB at Teesside University receives the excel spreadsheets from the Research Co-ordinators, creates a Master file, and saves a copy. She then removes the names from the excel spreadsheet, so that only screening numbers remain. She then sends this encrypted excel file (without names) to an independent statistician at Newcastle University (to be identified). The statistician will send JB a file showing the random allocations of these screening numbers to intervention or control. **This is only seen by JB**.
- JB will remerge the screening numbers and arm allocation that she receives from the statistician to the names and keep a record of which young person was allocated to intervention and control.
- In the meantime JB and the Alcohol and Public Health Team at Teesside University will be making up the intervention and control packs.
- Once JB has received the allocated list **only JB** will print intervention and control sheets, sticker all packs with relevant screening numbers and place them inside relevant packs depending on the allocation of participants. This will be double checked for accuracy by GW. **Only JB and GW will know to which arm young people were allocated**. Young peoples' names and school will be put on a sticker on the front of the envelope.

---

[1] Opt-out consent will already have been attained, prior to step 5.

- An envelope will be made for each young person. Inside the envelope will be the information sheet and assent forms and a sealed envelope. The sealed envelope will only be opened if the young person assents and this envelope will reveal the condition the young person has been allocated to.
- An assent log for each school will be made with the screening identification numbers, space to record the date and time of their appointment with the learning mentor and whether or not the young person agreed to take part.
- JB seals these envelopes and couriers them to sites outside Teesside University.
- Research Co-ordinators receive these sealed envelopes and **do not open them.**
- Research Co-ordinators take the sealed envelopes to the schools and give them to the learning mentors.
- Learning mentors see young people individually. The learning mentor asks the young person if they wish to take part. A note is made of this on the enclosed cover sheet and assent forms signed.
- Only then will the learning mentor open the second envelope and know which arm each young person has been allocated to. This information will be shared with the young person.
- The intervention or control condition is carried out by the learning mentors as relevant.
- At the end of the session, all relevant materials (e.g. control: assent update; intervention: assent update, case diary, worksheet) are placed back in the same envelope and sealed.
- The learning mentors give these sealed envelopes to the Research Co-ordinators.
- Research Co-ordinators **do not open** these envelopes and courier them back to Teesside University.
- At Teesside University, only JB and LA will open these envelopes, and both will make an electronic note on the database of whether each young person assented, and if yes took part. If any young people have not completed either an intervention or control (for e.g. due to absence from school), then JB and LA will need to liaise with Research Co-ordinators to let them know that they need to alert the learning mentors to ensure that when the young person is next present in school that they need to deliver either the intervention or control.
- **Only JB and LA (Lisa Anderson) will open these envelopes.**
- Over the next 11 months, JB and LA will finalise inputting a record of the intervention and control groups, and will make up packs for the 12-month follow up questionnaire.
- For those that assented to the trial, at the 12-month follow-up point, a repeat of the baseline questionnaire packs will be made. These will be couriered to sites.
- Research Co-ordinators will then take these envelopes into the schools. The young person will complete the 12-month questionnaire on their own, as at baseline. Once this is finished and in the envelope the Research Co-ordinator will go through the TLFB with the young person. The researcher will complete the TLFB. The TLFB will then be placed into the envelope and sealed in front of the young person. Researcher Co-ordinators will then take the envelopes back to the research site. **At no point do the research Co-ordinators ask which arm the young person was allocated to 12 months previously; young people should be discouraged from volunteering this information.**
- Research Co-ordinators will then input the data from these questionnaires to MACRO.
- Research Co-ordinators will courier the completed follow up questionnaires back to Teesside University.

12.2 Flowchart of study

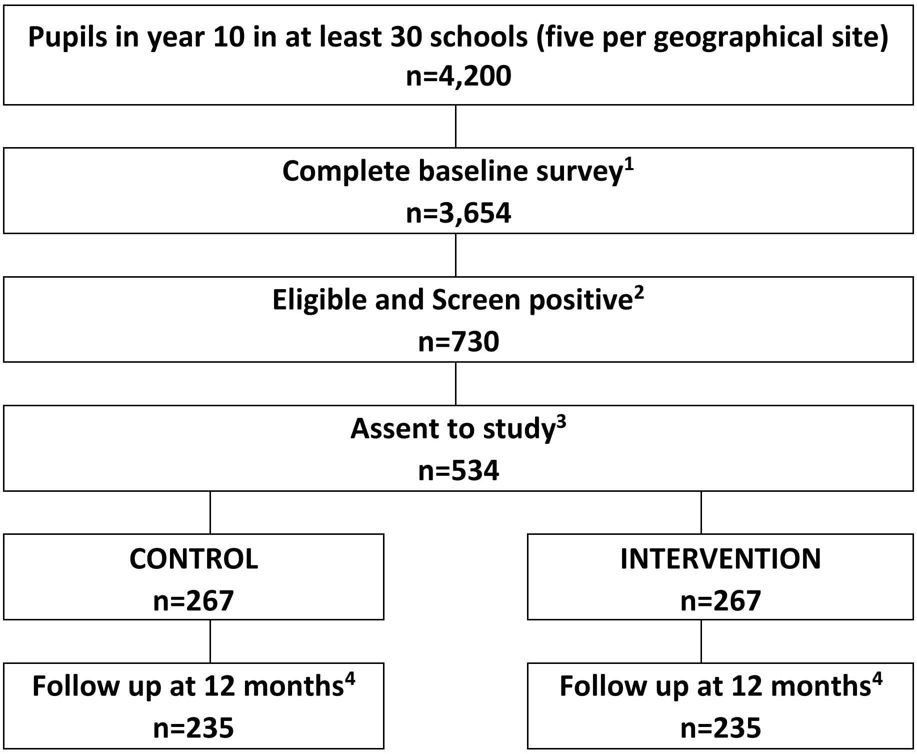

1. Complete baseline survey (87%); 2. Screen positive and leave name on questionnaire (20%); 3. Assent to study (80%); 4. 88% of those that assent to study. (%s assumed from pilot RCT (23)).

13. Screening, recruitment and assent

13.1 Screening and eligibility criteria

Screening will take place in the PSHE or registration class on a classroom by classroom basis.

13.2 Option to opt-out of screening

In advance of screening, all parents/caregivers will be informed by letter, sent by the school, that screening and the study will be taking place in their child's school. Parents will have the option to indicate that they do not wish for their child to be screened or considered for participation in the study at this stage by completing an opt-out form and returning to the co-ordinating research centre at Teesside University. Those young people whose parents have opted them out of the study will not complete the questionnaires and where possible will not be in the classroom at the time the survey takes place. We will work with the individual schools to ensure these children are given different tasks to do when the survey is taking place. Obtaining assent to take part in this manner is a method widely used in various national youth surveys of alcohol consumption and other health behaviours [2].

13.3 Screening for the trial

The teacher will introduce the questionnaires during a PSHE or registration class, making it clear to young people that completion of any identifiable information is not compulsory. A video-clip will be played to the entire class, in each school, to give instructions on completing

the questionnaires. This video-clip will only concentrate on the questionnaire completion and not on the topic of the questionnaires. Young people will be asked to voluntarily leave their name and class. Young people will have the option to not complete the questionnaire (indicative of lack of assent to screening from the young person), to complete the questionnaire anonymously, or to complete the questionnaire with their name and class. Each young person will place their completed questionnaires in a sealed envelope and return to the teacher. Teachers will be told not to open these envelopes. Individual responses will not be shared with the class teacher or learning mentor. The researcher will collect the sealed envelopes from the school. Those young people who have screened positively (Scoring 4 times or more frequently on the A-SAQ – see below) and have left their name will be eligible for the trial. Leaving a contact name did not create any issues in the pilot trial. Completed baseline questionnaires by trial participants will be used for the baseline measurements. Data from the whole year group (which includes everyone who has completed a questionnaire) will be written up as a journal article.

13.4 Data collection

13.4.1 Baseline data collection

The study envelope will contain a series of questionnaires (see section 9.1) including the study screening questionnaire: the A-SAQ 'In the last 12 months how often have you drunk more than 3 units of alcohol?' with the response options of 'Never; less than 4 times; 4 or more times but not every month; at least once a month but not every week; every week but not every day; every day'. Scoring 4 times or more frequently indicates a positive screen and eligibility for the trial. This score was shown in our pilot feasibility trial to be a methodologically robust approach to identifying the adolescent population who may benefit from an intervention [23]. The A-SAQ will be embedded within a larger questionnaire with items addressing a number of health and lifestyle topics.

13.4.2 Invitation to meet with learning mentors

- Returned survey questionnaires will be enclosed in a sealed envelope and taken back to the individual universities. The A-SAQ will be scored and a list of screening numbers and names of those that score positive and leave their name will be sent to the study administrator at Teesside University (see section 12.1 re randomisation procedure). The learning mentor will be given packs with names of potentially eligible young people. They will be given an assent log to complete of progress with potentially eligible young people. Learning mentors will invite young people for who they have an envelope for to a meeting with them in their office where they will open the relevant envelope for the young person. In the envelope will be an information leaflet and assent forms and a sealed envelope. Potential young people will be informed that participation is not compulsory and will be given the information leaflet to read. The assent form will ask for the first part of the young person's postcode as well as assent. The postcode information will be used to enable a stratified sample of young people who are asked to take part in the qualitative work. Once a young person has assented the second envelope will be opened which will state whether it is a control or intervention case. Until this point the learning mentor will not know which condition the young person has been allocated to.

13.5 Assent procedures

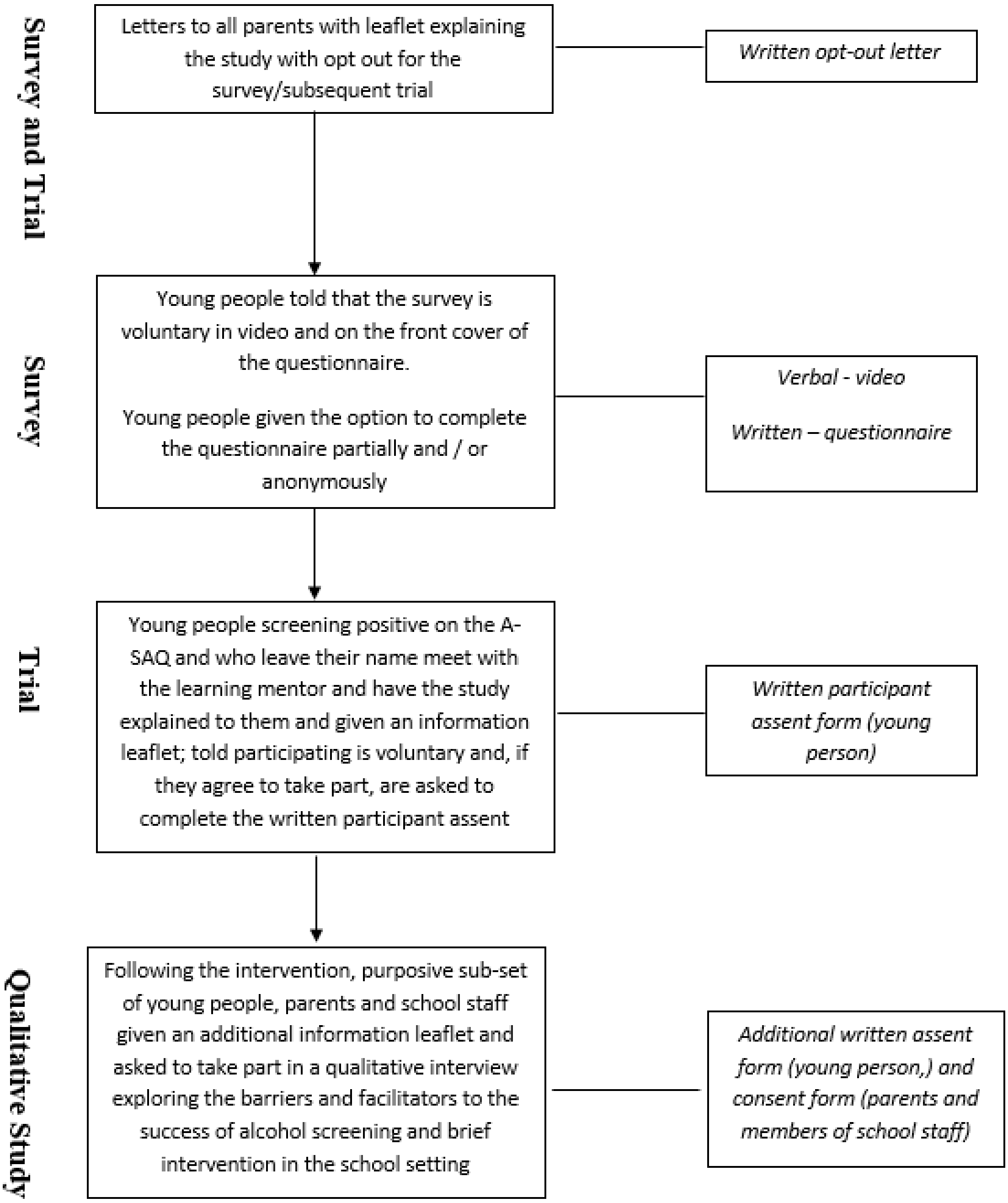

Nb: Ethical approval for qualitative work will be sought separately.

13.4.3 12-month follow-up

Follow-up will occur 12 months post intervention. All young people who come into the trial will be invited to meet with the project researcher (in the school setting) where they will be asked to complete the same battery of questionnaires used at baseline. If a young person involved in the study has moved to another school attempts will be made to contact them there to complete the questionnaires. The researcher will be blinded to the condition the young person was allocated to. The TLFB (including the primary outcome measure of total alcohol consumption consumed) will also be completed face-to-face in schools with the researcher in order to limit bias in the results. All trial participants will be given a cinema

voucher to compensate them for the time involved in the study [46]. Trial participants' baseline and follow-up questionnaires will be linked with a unique ID (the screening number). All participants will be asked by the researcher at follow-up whether they are willing to be contacted by a researcher who may be the same individual or another researcher for an in-depth interview.

13.4.4 Intervention fidelity

An important measure of process relates to how the intervention is conducted which will also help to understand barriers and facilitators to rolling out the intervention. Each learning mentor will have one simulated intervention with another learning mentor or the research co-ordinator, recorded post training (competency check). Learning mentors will only be allowed to go 'live' if the research co-ordinator believes they are competent. Of these sessions, at least 80% will be recorded and further checked by an expert rater (RM). Furthermore twenty per cent of randomly selected cases in the trial will be audio taped and transcribed and assessed for treatment fidelity by one independent expert rater from the research team (RM), with any discrepancies discussed with a second expert rater (EG); using the BECCI rating scale [47]. The BECCI scale is scored 0-4 with a score of two or more being accepted as acceptable as used in previous studies [22-25]. The young people will provide assent for this recording to take place. As the recording and analysis of the delivery of the intervention sessions forms part of the employment contract of the learning mentors, formal consent is not required.

14. Project timetables and milestones

	Months	
-6-0	03/15-08/15	Protocol development, ethics application, recruitment of staff
1-3	09/15-11/15	Study set up and training of Research Co-ordinators
4	12/15	Training of learning mentors, and provision of opt-out letters to parents
4	12/15	Baseline survey and screening
5-7	01/16-03/16	Case recruitment (control/intervention)
7-9	03/16-05/16	Staff interviews
17-19	01/17-03/17	12 month follow-ups with trial participants
19-20	03/17-04/17	Young people interviews
7-26	04/16-10/17	Qualitative analysis
6-26	05/16-10/17	Data inputting and analysis of RCT
24-28	08/17-12/17	Writing of final report
27-28	11/17-12/17	Dissemination

15. Statistical considerations

15.1 Sample size calculation

Using estimates from the pilot trial (mean year group size = 210, 87% completing baseline survey, 20% being positive on A-SAQ and leaving contact details, 80% recruited to trial and 88% providing data at 12 months follow-up), the sample size has been calculated to have a 90% power to detect a standardized difference of 0.3 (which equates to a ratio of 1.5 in geometric means in total alcohol units in 28 days) using a significance level of 5%. Follow-up data will be required on 235 children per arm. The number of young people in 20 schools (5 per region) however means that this number will be increased to 235 in each arm at follow-

up. Anticipated numbers at each point of the study are illustrated in the flowchart in section 12.2.

15.2 Analysis

15.2.1 Baseline data

Descriptive statistics (comparisons of percentages, means or medians as appropriate) will be used to report the pupil-level baseline data, and extent of intervention received between those allocated to the two trial arms.

15.2.2 Primary outcome

The researchers will be blind to the randomisation condition. The primary outcome is derived from the 28-day TLFB (units of alcohol consumed in period). The primary effectiveness analysis will be by intention-to-treat. Multiple linear regression will be used to compare the primary outcomes between the two randomisation groups at 12 months, adjusting for any imbalance in key covariates.

15.2.3 Secondary outcomes

The secondary outcomes will be analysed in a similar manner. Comparisons of means will be presented as mean differences or ratios of geometric means (if a logarithmic transform is necessary for skewed data) with 95% C.I. Odds ratios and 95% C.I.'s will be presented for binary outcomes. Exploratory analyses will also be undertaken, for example, to examine differences in outcome by gender, deprivation and extent of intervention received, though there is limited power to investigate these comparisons. We will consider any difference in attrition rates, and any non-randomness of the attrition, when comparing outcomes between the two groups.

15.2.4 Interim analyses

There are no planned interim analyses, other than descriptive analysis to report on recruitment.

15.2.5 Missing data

The pattern and extent of missing observations because of loss to follow-up will be examined to investigate both the extent of missingness, and whether it is missing at random or is informative. Unless specified by the scale developers, where no more than 20% of questions are missing or uninterpretable on specific scales, the score will be calculated by using the mean value of the respondent specific completed responses on the rest of the scale to replace the missing items. The use of appropriate multiple imputation techniques will be considered.

16. Health economics

The economic component will include both a within trial cost-utility and cost-consequence analysis and, as described below, a model based analysis taking the perspective of the UK public sector (NHS, educational, social, and criminal services). The cost-utility analysis will use measures of effectiveness limited to health related quality of life as measured by EQ-5D Y. The cost-consequence analysis will take the same perspective for costs but will present these alongside all of the primary and secondary measures of effectiveness outlined in section 9.3. The follow-up for the within trial analyses will be 12 months so discounting will not be conducted. For the model based analysis the time horizon will be longer (potentially up to the

participant's life time) and costs and effects will be discounted at 1.5%, the UK recommended rate for public health interventions [48] with, a sensitivity analysis used to explore the impact of higher (and lower) discount rates.

16.1 Within trial analysis cost-utility and cost-consequence analyses

For each trial participant the use of health, educational, criminal, and social care services will be elicited using the S-SUQ administered at baseline (with a recall period of 3 months) and 12 months. Further cost data will come from the learning mentor time case diaries completed by the learning mentors for each contact. Costs for healthcare and social services will be obtained from standard sources such as NHS reference (www.gov.uk), the British National Formulary [49] for medications, Unit costs of Health and Social Care [50] for contacts with primary care. Further data will come from the study centres themselves. Data on the use of educational services will be elicited via the questionnaire. As part of the pilot trial we confirmed with the expert group the type of services relevant to collect. However based on lessons learned from the pilot additional questions related to days missed from school/ truancy have been added to the questionnaire.

Learning mentor training costs will be included and will need to be apportioned according to scaled up practice. This will be informed by data from the training conducted as part of the trial and through expert opinion. The time of educational staff will be sought through a parallel costing exercise in which these staff will be asked to provide information on the impact of the intervention on their workload. With respect to learning mentors, a detailed proforma was developed and tested in the pilot to capture resource use and this new tool will be used in this study. With respect to school building and other large capital items, the opportunity cost will be considered. Some resources (e.g. buildings) will exist with or without the intervention and the intervention may not displace any other activity. In this circumstance the opportunity cost of the building would be zero. However, costs might be incurred in terms of heat, power and light and these data will be captured using standard costing methods [51]. For each participant, measures of use of resources will be combined with unit costs to provide a cost for that participant. We anticipate that the price year adopted for the base case analysis will be 2017 when the final analysis is conducted.

Like in the pilot trial we will use the European Quality of Life Five Dimension – Youth (EQ-5D-Y) was used. The EQ-5D Y will be administered at baseline and 12 months with UK population tariffs [52] used. Health state utilities from the EQ-5D Y will then be used to estimate QALYs using the area under the curve approach [38].

Data on costs and QALYs will be used to estimate mean cost and QALYs for the intervention and control groups. The cost and QALY data will then be used to estimate incremental costs and QALYs and incremental costs per QALY gained. These data will be presented as point estimates and bootstrapping techniques will be used to estimate the statistical imprecision surrounding them. The results of this stochastic analysis will be presented as cost and QALY plots and as cost-effectiveness acceptability curves. Linear interpolation between time points will be used, assuming the change happens at the end of the time point.

The cost-consequence analysis will present the cost data and effects data in the form of balance sheets. In the balance sheets the interventions will be presented in a series of pairwise comparisons with data on costs and effects presented as pros and cons for an

experimental intervention compared with a control. Thus, the approach can capture wider effects than those captured by measures of cost or quality of life. The principle underpinning a balance sheet is that the analyst should seek to capture all costs and benefits no matter on whom they may fall; the same principles underpinning a cost-benefit analysis [53]. This approach has been used in prior evaluations as a way of integrating both quantitative and qualitative findings into a single assessment [54, 55].

16.2 Model based analysis

In an economic evaluation the time horizon should be sufficiently long enough to capture all costs and benefits of relevance. Ideally, within a trial setting the data collection period would be sufficiently long enough to capture all relevant costs and benefits. Such a proposal would significantly increase costs, increase burden on participants, and costs and benefits in the longer term may be subject to a host of exogenous factors. Hence, the longer term collection of data within a trial setting may not produce reliable data on longer term outcomes. In the absence of longer term trial data, longer term data from the literature will be considered. The economic model based analysis, most likely taking the form of a state transition model, will be conducted if it is plausible that extrapolation over a longer time horizon could change the within trial based analyses. For example, if at the end of the 12 month follow-up, the QALY gain is not of sufficient magnitude to justify the cost to society, modelling can illustrate whether the eventual long-term gain becomes more worthwhile. The model will be constructed following guidelines for best practice in economics modelling [56, 57]. The use of services will be modelled and the costs of these events will be based on data from the trial and, where necessary, supplemented by focused searches of the literature and health economic databases, (National Health Service Economic Evaluations Database (NHS EED) and the CEA Registry. As already noted both costs and outcomes will be discounted at 1.5% in the base case analyses. The model will be used to produce estimates of costs, QALYs, incremental cost per QALY gained, and cost-consequences. The model will be probabilistic and distributions will be attached to all parameters, the shape and type of distribution will depend upon the data available and recommendations for good practice in modelling [56]. The results will also be presented as point estimates, and for the cost-consequence analysis 95% confidence intervals. For the cost-utility analysis, data will be presented as plots of costs and QALYs derived from the probabilistic analysis and cost-effectiveness acceptability curves. Deterministic sensitivity analyses to explore other uncertainties will also be conducted.

17. Qualitative work

Separate ethical approval will be sought for the qualitative work.

18. Triangulation

Once the quantitative and qualitative elements of the study have been carried out and analysed separately they will be brought together at the ‘analysis/interpretation’ phase which is a process often described as 'triangulation' [58]. In our study, data will be reconciled by adopting a model which relies on the principle of complementarity [59]. Within this approach it is explicitly recognised that qualitative and quantitative methods may be used to examine different aspects of an overall research question [58].

19. Compliance and withdrawal

19.1 Assessment of compliance

Where feasible, visits to the individual school in the geographical sites will happen at least once every two weeks with telephone calls if necessary in between. These study visits will be conducted by site Research Co-ordinators.

19.2 Withdrawal of participants

Young people who do not leave their name on the questionnaire will not be able to be identified post completion and therefore their data cannot be withdrawn if requested. For the trial, participants have the right to withdraw from the trial at any time for any reason, and without giving a reason. The investigators also have the right to withdraw participants from the study intervention if s/he judges this to be in the participant's best interests. It is understood by all concerned that an excessive rate of withdrawals can render the study uninterpretable; therefore, unnecessary withdrawal of participants should be avoided. Should a participant decide to withdraw from the study, all efforts will be made to report the reason for withdrawal as thoroughly as possible.

There are two withdrawal options:

1. Withdrawing completely (i.e. withdrawal from both the study intervention and provision of follow-up data)
2. Withdrawing partially (i.e. withdrawal from study intervention but continuing to provide follow-up data by completing 12-month follow-up questionnaires).

Assent will be sought from participants choosing option 1 to retain data collected up to the point of withdrawal. Participants will be asked if they would be happy for the reason for the decision to withdraw to be recorded.

20. Data monitoring, quality control and quality assurance

This is a low risk trial and major safety data are not anticipated. As agreed by Newcastle University/NIHR PHR a TSC will be set up as well as a separate Data Monitoring and Ethics Committee (DMEC). Both will occur with independent members meeting in closed session. The groups will also take responsibility for monitoring study conduct and data collected will be performed by central review to ensure the study is conducted in accordance with GCP. The main areas of focus will include assent/consent, data quality and essential documents in the study. The TSC will consist of Professor Matthew Hickman as Chair, an independent school representative, independent statistician, the CI of the study (DNB); the Project Manager (EG); the study statistician (DH) and other members of the TSG as appropriate. Following the initial pre-study meeting, the TSG will meet annually. Their role is to monitor progress and supervise the trial to ensure it is conducted to high standards in accordance with the protocol, the principles of GCP, relevant regulations and guidelines and with regard to participant safety. The purpose of this committee will be to monitor efficacy and safety endpoints, although only independent members may have access to unblinded study data. A written charter will be agreed and used by the TSC. All monitoring findings will be reported and followed up with the appropriate persons in a timely manner. The DMEC will take responsibility for the ethical compliance of the trial and will meet once yearly prior to the TSG meetings.

The study may be subject to inspection and audit by Newcastle University under their remit as sponsor, and other regulatory bodies to ensure adherence to GCP. The investigators/ institutions will permit trial-related monitoring, audits, ethical committee review and regulatory inspection(s), providing direct access to source data/documents.

Table of events

**Time**	**Visit 1 Initial Screening**	**Visit 2 Baseline visit Confirmation of eligibility and Randomisation**	**12 month follow-up 12 months post baseline (+/- 10 weeks)**
Study questionnaire completion	**X**		**X**
Study discussion / Informed assent		**X**	
Informed of randomisation allocation		**X**	
28 day TLFB questionnaire			**X**

The quality and retention of study data will be the responsibility of Professor Dorothy Newbury-Birch, who will act as data custodian for the study. All study data will be retained in accordance with the Data Protection Act (1998), the Directive on GCP (2005/28/EC), sponsor and local policy.

21. Adverse event monitoring and reporting

Due to the nature of the study it is not expected that participants will experience any adverse events/serious adverse events during the study. In the event that the participant reports an event related to the study during a study visit this will be reported on the adverse event/ harms case report form and entered into MACRO.

22. Ethics and regulatory issues

As participants are not being recruited from the NHS, the proposed research will not require NHS ethical approval but we will seek multi-site ethical approval from Teesside University ethics committee, which covers all non-NHS studies carried out at the University. Information sheets will be provided to all eligible subjects and written informed assent/consent obtained prior to any study procedures.

23. Research governance

Newcastle University will be the nominated sponsor of the research and will hold the award. Professor Newbury-Birch will be the Chief Investigator based at Teesside University together with the Project Manager and North East staff relating to the study. The Research Co-ordinators and Project Manager will meet weekly (by Skype) to progress the study (Working Group). Other investigators will be invited to attend meetings when necessary. The study will have a TMG, which will consist of the Chief Investigator, co-applicants, Project Manager,

Research Co-ordinators, researchers and CTU staff involved in the study as well as two lay members (to be identified). Professor Eilish Gilvarry who chaired the pilot study TMG will chair this group. Further to this we will set up an independent TOC (see section 18) with membership in accordance with NIHR guidelines. The project will be subject to the requirements of the Data Protection Act 1998 and the Freedom of Information Act 2000 and other relevant UK and European legislation relevant to the conduct of clinical research. The project will be managed and conducted in accordance with the MRCs Guidelines on Good Clinical Practice in Clinical Trials (www.mrc.ac.uk), which will include compliance with national and international regulations on the ethical involvement of participants in clinical research (including the Declaration of Helsinki). Newcastle Clinical Trials Unit Standard Operating Procedures will be followed.

All data for the study will be held in a secure environment identified by a screening ID. Master registers containing participant identifiable information and participant identification numbers will be stored in a secure area separate from the majority of data. Remote electronic data capture and data management will be conducted by Newcastle Clinical Trials Unit using Elsevier's MACRO. All staff employed on the project will be employed by academic organisations and subject to the Terms and Conditions of Service and contracts of employment of the employing organisations. The project will use standardised research and clinical protocols and adherence to the protocols will be monitored by the Trial Steering Committee.

All trial data will be identified using a unique trial identification number (the screening number). No personally identifiable information will be held beyond the final 12-month follow-up. Analytical datasets will not contain any participant identifiable information. Anonymised hard-copy data will be retained for a period of five years following the end of the trial. Electronic data will be kept for 10 years following the end of the trial.

24. Confidentiality

Personal data will be regarded as strictly confidential. To preserve anonymity, any data relating to the questionnaire leaving the sites will be anonymised and will identify participants with their screening number. The study will comply with the Data Protection Act, 1998. All study records and Investigator Site Files will be kept at site in a locked filing cabinet with restricted access. All data will be sent to the co-ordinating centre (Teesside University) by secure courier where it will be kept in a locked filing cabinet with restricted access. Names of those in the trial will be sent off site by secure email /courier by site study Research Co-ordinators to the administrative assistant at the co-ordination centre (Teesside University) and will be couriered separately from the questionnaire responses.

25. Insurance and finance

Indemnity in respect of potential liability arising from negligent harm relating to design and conduct of the research is provided by Teesside University for those protocol authors who have their substantive contracts of employment with Teesside University. Indemnity in respect of potential liability arising from negligent harm relating to design and conduct of the research is provided by Newcastle University for those protocol authors who have their substantive contracts of employment with Newcastle University.

Indemnity in respect of potential liability arising from negligent harm relating to management of the research is provided by the Sponsor.

This is a non-commercial study and there are no arrangements for non-negligent compensation. NIHR Public Health Research Programme is funding the study.

26. Study report/publications

The data will be the property of the Chief Investigator and Co-applicants. Publication will be the responsibility of the Chief Investigator. It is planned to publish this study in peer review articles and to present data at national and international meetings. Results of the study will also be reported to the Sponsor and Funder, and will be available on their web site. All manuscripts, abstracts or other modes of presentation will be reviewed by the TOC and funder prior to submission. Individuals will not be identified from any study report. Participants will be informed about the results at the end of the study, including a lay summary of the results if requested.

27. References

1. Hibbell, B., et al., *The 2011 ESPAD Report: Substance Use Among Students in 36 European Countries.* 2012.
2. Fuller, E. and Et al, *Smoking, drinking and drug use among young people in England in 2013*. 2014, NatCen Social Research: London.
3. Zucker, R., et al., *Developmental perspective on underage alcohol use. Developmental processes and mechanisms 0-10.* Alcohol Research and Health, 2009. **32**(1): p. 16-29.
4. Windle, M., et al., *Transitions Into Underage and Problem Drinking Summary of Developmental Processes and Mechanisms: Ages 10–15.* Alcohol Research and Health, 2009. **32**(1): p. 30-40.
5. Brown, S., et al., *Underage Alcohol Use Summary of Developmental Processes and Mechanisms: Ages 16–20.* Alcohol Research and Health, 2009. **32**(1): p. 41-52.
6. Newbury-Birch, D., et al., *The impact of alcohol consumption on young people: A review of reviews*. 2009, Department of Children Schools and Families.
7. Squeglia, L.M., et al., *Brain response to working memory over three years of adolescence: Influence of initiating heavy drinking.* Journal of Studies on Alcohol and Drugs, 2012. **73**(5): p. 749.
8. Rodham, K., et al., *Adolescents' perception of risk and challenge: A qualitative study.* Journal of Adolescence, 2006. **29**: p. 261-72.
9. MacArthur, G., et al., *Patterns of alcohol use and multiple risk behaviour by gender during early and late adolescence: the ALSPAC cohort.* Journal of Public Health, 2012. **34**(Suppl 1): p. i20-i30.
10. NHS Choices. *The risks of drinking too much*. 05/08/2014]; Available from: www.nhs.uk/Livewell/alcohol/Pages/Effectsofalcohol.aspx.
11. McGue, M., et al., *Origins and Consequences of Age at First Drink. Associations with Substance-use Disorders, Dishinbitory Behavior and Psychopathology, and P3 Amplitude.* Alcoholism, Clinical and Experimental Research, 2001. **25**(8): p. 1156-1165.
12. Donaldson, L., *Guidance on the consumption of alcohol by children and young people*. 2009, Department of Health: London.
13. National Institute for Clinical Excellence, *Interventions in schools to prevent and reduce alcohol use among children and young people*. 2007, NICE: London.

14. Foxcroft, D. and A. Tsertsvadze, *Universal shool-based prevention programs for alcohol misuse in young people*, in *Cochrane Database of Systematic Reviews*. 2011.
15. World Health Organization, *Alcohol in the European Union. consumption, harm and policy approaches*, ed. P. Anderson, L. Moller, and G. Galea. 2012: WHO Regional Office for Europe.
16. Kaner, E., et al., *The effectiveness of brief alcohol interventions in primary care settings: a comprehensive review.* Drug and Alcohol Review, 2009. **28**: p. 301-323.
17. Bandura, A., *Social learning theory*. 1997, Englewood Cliffs, NJ: Prentice-Hall.
18. Bien, T.H., W.R. Miller, and J.S. Tonigan, *Brief interventions for alcohol problems: a review.* Addiction, 1993. **88**(3): p. 315-335.
19. Kaner, E., et al., *Effectiveness of brief alcohol interventions in primary care populations.* Cochrane Database of Systematic Reviews, 2007. **Issue 2**.
20. Miller, W. and V. Sanchez, *Motivating Young Adults for Treatment and Lifestyle Change*. 1993, Notre Eame: University of Notre Dame Press.
21. Rollnick, S., P. Mason, and C. Butler, *Health Behaviour Change: A guide for practitioners*. 1999, Edinburgh: Churchill Livingstone.
22. Kaner, E., et al., *Effectiveness of screening and brief alcohol intervention in primary care (SIPS trial): pragmatic cluster randomised controlled trial.* BMJ, 2013. **346**: p. 1-14.
23. Newbury-Birch, D., et al., *A pilot feasibility cluster randomised controlled trial of screening and brief alcohol intervention to prevent hazardous drinking in young people aged 14-15 in a high school setting (SIPS JR-HIGH)*, N.P.H.R.P. Report, Editor. 2014.
24. Drummond, C., et al., *The effectiveness of alcohol screening and brief intervention in emergency departments: a multicentre pragmatic cluster randomized controlled trial.* PLOS ONE, 2014. **9**(6): p. e99463.
25. Newbury-Birch, D., et al., *Alcohol screening and brief interventions for offenders in the probation setting (SIPS Trial): a pragmatic multicentre cluster randomised controlled trial.* Alcohol & Alcoholism, 2014. **In Press**.
26. Department of Health, *The Annual Report of the Chief Medical Officer 2012: Our Children Deserve Better: Prevention Pays*. 2013, Department of Health: London.
27. Yuma-Guerrero, P., et al., *Screening, Brief Intervention, and Referral for alcohol Use in Adolescents: A Systematic Review.* Pediatrics, 2012. **130**: p. 115-122.
28. Tripodi, S.J., et al., *Interventions for reducing adolescent alcohol abuse: A meta-analytic review.* Archives of Pediatrics and Adolescent Medicine, 2010. **164 (1)**: p. 85-91.
29. Carney, T. and B. Myers, *Effectiveness of early interventions for substance-using adolescents: findings from a systematic review and meta-analysis.* Substance Abuse Treatment, Prevention, and Policy, 2012. **7**(1): p. 25.
30. O'Neil, S., et al., *Brief intervention to prevent hazardous drinking in young people aged 14-15 in a high school setting (SIPS JR-HIGH): study protocol for a randomized controlled trial.* Trials, 2012. **13**(166).
31. Babor, T., et al., *AUDIT, The Alcohol Use Disorders Identification Test, guidelines for use in primary health care*. 1989, World Health Organisation: Geneva.
32. Reinert, D.F. and J.P. Allen, *The Alcohol Use Disorders Identification Test: An Update of Research Findings.* Alcoholism: Clinical & Experimental Research, 2007. **Vol. 31**(No. 2): p. pp. 185-199.
33. White, H. and H. Labouvie, *Towards the assessment of adolescent problem drinking.* Journal of studies of Alcohol, 1989. **50**(30): p. 7.

34. Cooper, M., *Motivations for alcohol use among adolescents: Development and validation of a four-factor model.* Psychological Assessment, 1994. **6**: p. 117-128.
35. UKATT Research Team, *Cost effectiveness of treatment for alcohol problems: findings of the randomised UK alcohol treatment trial (UKATT).* British Medical Journal, 2005. **331**(7516): p. 544.
36. NHS Health Scotland and University of Warwick, *Warwick-Edinburgh Mental Well-Being Scale (WEMWBS)*. 2006: Scotland.
37. Tennant, R., et al., *The Warwick-Edinburgh Mental Well-being Scale (WEMWBS): development and UK validation.* Health and Quality of Life Outcomes, 2007. **5**: p. 63.
38. Euroquol, *EuroQuol-a new facility for the measurement of health-related quality of life.* Health Policy, 1990. **16**: p. 199 - 208.
39. Sobell, L. and M. Sobell, *Alcohol Timeline Followback Users' Manual*. 1995, Toronto, Canada: Addiction Research Foundation.
40. Segrott, J., et al., *Preventing substance misuse: study protocol for a randomised controlled trial of the Strengthening Families Programme 10–14 UK (SFP 10–14 UK).* BMC Public Health, 2014. **14**(49): p. 1-21.
41. Talbot, S. and T. Crabbe, *Binge drinking: young people's attitude and behaviour*, P. Futures, Editor. 2008, Crime Concern: London.
42. Nelson, M., et al., *Seventh annual survey of take up of school lunches in England* 2012, Children's Food Trust.
43. Office for National Statistics, *Ethnicity and National Identity in England and Wales 2011*. 2012.
44. Medical Research Council, *Developing and evaluating complex interventions: new guidance*. 2008: London.
45. Kaner, E.F.S., et al., *A RCT of three training and support strategies to encourage implementation of screening and brief alcohol intervention by general practitioners.* British Journal of General Practice, 1999. **49**: p. 699-703.
46. Brueton, V., et al. *Systematic Review of Strategies to Reduce Attrition in Randomised Trials*. in *The Society for Clinical Trials Annual Meeting*. 2013. Boston: Clinical Trials.
47. Lane, C., et al., *Measuring adaptations of motivational interviewing: The development and validation of the Behaviour Change Counselling Index (BECCI),.* Patient Education and Counselling 2005. **56**: p. 166-173.
48. National Institute for Clinical Excellence, *Guide to the methods of technology appraisal*. 2013: London.
49. British Medical Association and Royal Pharmaceutical Society, *British National Formulary Edition 65*. 2013.
50. Curtis, L., *Unit Costs of Health and Social Care 2012*, PSSRU, Editor. 2013, University of Kent: Canterbury.
51. Drummond, M., G. Stoddart, and G. Torrance, *Methods for the Economic Evaluation of Health Care Programmes*. 2005, Oxford: Oxford University Press.
52. Kind, P., G. Hardman, and S. Macran, *UK population norms for EQ-5D*. 1999, Centre for Health Economics: University of York.
53. McIntosh, E., *Economic evaluation of guideline implementation strategies*, in *Changing professional practice: Theory and practice of clinical guidelines implementation*, T. Thorson and M. Makela, Editors. 1999, Danish Institute for Health Services: Copenhagen.
54. Hoddinott, P., et al., *Process evaluation for the FEeding Support Team (FEST) randomised controlled feasibility trial of proactive and reactive telephone support for*

breastfeeding women living in disadvantaged areas. BMJ Open, 2012. **2**(2): p. e001039.

55. Hoddinott, P., et al., *The FEeding Support Team (FEST) randomised, controlled feasibility trial for proactive and reactive telephone support for breastfeeding women living in disadvantaged areas.* BMJ Open, 2012. **2**(2): p. e000652.
56. Kaltenthaler et al. *NICE DSU Technical Support Document 13* Available from: http://www.nicedsu.org.uk.
57. Caro, J., et al., *Modeling good research practices—overview: a report of the ISPOR-SMDM modeling good research practices task force-1.* Value Health, 2012. **15**(796-803).
58. O'Cathain, A., E. Murphy, and J. Nicholl, *Three techniques for integrating data in mixed methods studies* British Medical Journal, 2010. **341**: p. 1147-1150.
59. Moffatt, S., et al., *Using quantitative and qualitative data in health services research – what happens when mixed method findings conflict?* BMC Health Services Research, 2006. **6**(28): p. 1-10.
