# Supplementary material for: Multicentre individual randomised controlled trial of screening and brief alcohol intervention to prevent risky drinking in young people aged 14–15 in a high school setting (SIPS JR-HIGH): study protocol
Source: BMJ Open. 2016 Dec 23;6(12):e012474. doi: 10.1136/bmjopen-2016-012474 (PMC5223663; doi:10.1136/bmjopen-2016-012474)
Supplement: supplementary appendix [file bmjopen-2016-012474supp_appendix2.pdf]

A multi-centre individual randomised controlled trial of screening and brief alcohol intervention to prevent risky drinking in young people aged 14-15 in a high school setting (SIPS JR-HIGH): **Pupil assent form**

A multi-centre individual randomised controlled trial of screening and brief alcohol intervention to prevent risky drinking in young people aged 14-15 in a high school setting (SIPS JR-HIGH): Pupil Assent Form

Please read each of the following statements and place your initials in the box if you agree with the statement. If you have initialled every statement please write your name and the date below.

1. I have had a chance to read the participant information leaflet dated 17.07.2015 (version 2.0) for the above study. ☐
2. Someone else has explained this project to me. ☐
3. I have had the opportunity to ask all of the questions I want about this study and they have been answered to my satisfaction. ☐
4. I understand that taking part is voluntary and that I am free to change my mind at any time without giving a reason and without my education, services from school and legal rights being affected. ☐
5. I understand that data from my school records may be looked at by members of the research team if it is relevant to my taking part in this research. ☐
6. I understand that any data created from this study will be held in a locked filing cabinet for five years after the trial, when the paper copies of the data will be destroyed. Electronic data will be stored on password protected computers for ten years. All data collected will be anonymised and kept confidential. ☐
7. I understand I will be contacted for follow up in 12 months and my data will be kept until this point. ☐
8. I agree to my session being recorded if asked. ☐
9. I understand that I may be asked to take part in an interview on my experience of taking part in this study. ☐
10. **I agree to take part in this study. I am aware that a copy of this consent form will be provided to me for my records.** ☐

____________________	____________________	____________________
Name of Participant	Signature	Date
____________________	____________________	____________________
Name of Witness	Signature	Date

Participant Postcode

The participant, school and research co-ordinating centre at Teesside University will have a copy of this form.

NHS National Institute for Health Research

This project was funded by the National Institute for Health Research PHR (project number 13/177/002
